# Assessing the impact of anthropogenic pollution on isoprene-derived secondary organic aerosol formation in PM_2.5_ collected from the Birmingham, Alabama, ground site during the 2013 Southern Oxidant and Aerosol Study

**DOI:** 10.5194/acp-16-4897-2016

**Published:** 2017

**Authors:** Weruka Rattanavaraha, Kevin Chu, Sri Hapsari Budisulistiorini, Matthieu Riva, Ying-Hsuan Lin, Eric S. Edgerton, Karsten Baumann, Stephanie L. Shaw, Hongyu Guo, Laura King, Rodney J. Weber, Miranda E. Neff, Elizabeth A. Stone, John H. Offenberg, Zhenfa Zhang, Avram Gold, Jason D. Surratt

**Affiliations:** 1 Department of Environmental Sciences and Engineering, Gillings School of Global Public Health, The University of North Carolina at Chapel Hill, Chapel Hill, NC, USA; 2 Atmospheric Research & Analysis, Inc., Cary, NC, USA; 3 Electric Power Research Institute, Palo Alto, CA, USA; 4 Earth and Atmospheric Science, Georgia Institute of Technology, Atlanta, GA, USA; 5 Department of Chemistry, University of Iowa, Iowa City, IA, USA; 6 Human Exposure and Atmospheric Sciences Division, United States Environmental Protection Agency, Research Triangle Park, NC, USA; a now at: Earth Observatory of Singapore, Nanyang Technological University, Singapore; b now at: Michigan Society of Fellows, Department of Chemistry, University of Michigan, Ann Arbor, MI, USA

## Abstract

In the southeastern US, substantial emissions of isoprene from deciduous trees undergo atmospheric oxidation to form secondary organic aerosol (SOA) that contributes to fine particulate matter (PM_2.5_). Laboratory studies have revealed that anthropogenic pollutants, such as sulfur dioxide (SO_2_), oxides of nitrogen (NO_*x*_), and aerosol acidity, can enhance SOA formation from the hydroxyl radical (OH)-initiated oxidation of isoprene; however, the mechanisms by which specific pollutants enhance isoprene SOA in ambient PM_2.5_ remain unclear. As one aspect of an investigation to examine how anthropogenic pollutants influence isoprene-derived SOA formation, high-volume PM_2.5_ filter samples were collected at the Birmingham, Alabama (BHM), ground site during the 2013 Southern Oxidant and Aerosol Study (SOAS). Sample extracts were analyzed by gas chromatography-electron ionization-mass spectrometry (GC/EI-MS) with prior trimethylsilylation and ultra performance liquid chromatography coupled to electrospray ionization high-resolution quadrupole time-of-flight mass spectrometry (UPLC/ESI-HR-QTOFMS) to identify known isoprene SOA tracers. Tracers quantified using both surrogate and authentic standards were compared with collocated gas- and particle-phase data as well as meteorological data provided by the Southeastern Aerosol Research and Characterization (SEARCH) network to assess the impact of anthropogenic pollution on isoprene-derived SOA formation. Results of this study reveal that isoprene-derived SOA tracers contribute a substantial mass fraction of organic matter (OM) (~ 7 to ~ 20 %). Isoprene-derived SOA tracers correlated with sulfate (${\mathrm{SO}}_{4}^{2-}$) (*r*^2^ = 0.34, *n* = 117) but not with NO_*x*_. Moderate correlations between methacrylic acid epoxide and hydroxymethyl-methyl-α-lactone (together abbreviated MAE/HMML)-derived SOA tracers with nitrate radical production (P[NO_3_]) (*r*^2^ = 0.57, *n* = 40) were observed during nighttime, suggesting a potential role of the NO_3_ radical in forming this SOA type. However, the nighttime correlation of these tracers with nitrogen dioxide (NO_2_) (*r*^2^ = 0.26, *n* = 40) was weaker. Ozone (O_3_) correlated strongly with MAE/HMML-derived tracers (*r*^2^ = 0.72, *n* = 30) and moderately with 2-methyltetrols (*r*^2^ = 0.34, *n* = 15) during daytime only, suggesting that a fraction of SOA formation could occur from isoprene ozonolysis in urban areas. No correlation was observed between aerosol pH and isoprene-derived SOA. Lack of correlation between aerosol acidity and isoprene-derived SOA is consistent with the observation that acidity is not a limiting factor for isoprene SOA formation at the BHM site as aerosols were acidic enough to promote multiphase chemistry of isoprene-derived epoxides throughout the duration of the study. All in all, these results confirm previous studies suggesting that anthropogenic pollutants enhance isoprene-derived SOA formation.

## 1 Introduction

Fine particulate matter, suspensions of liquid or solid aerosol in a gaseous medium that are less than or equal to 2.5 μm in diameter (PM_2.5_), play a key role in physical and chemical atmospheric processes. They influence climate patterns both directly, through the absorption and scattering of solar and terrestrial radiation, and indirectly, through cloud formation (Kanakidou et al., 2005). In addition to climatic effects, PM_2.5_ has been demonstrated to pose a human health risk through inhalation exposure (Pope and Dockery, 2006; Hal-lquist et al., 2009). Despite the strong association of PM_2.5_ with climate change and environmental health, there remains a need to more fully resolve its composition, sources, and chemical formation processes in order to develop effective control strategies to address potential hazards in a cost-effective manner (Hallquist et al., 2009; Boucher et al., 2013; Noziere et al., 2015).

Atmospheric PM_2.5_ is comprised in a large part (up to 90 % by mass in some locations) of organic matter (OM) (Carlton et al., 2009; Hallquist et al., 2009). OM can be derived from many sources. Primary organic aerosol (POA) is emitted from both natural (e.g., fungal spores, vegetation, vegetative detritus) and anthropogenic sources (fossil fuel and biomass burning) prior to atmospheric processing. As a result of large anthropogenic sources, POA is abundant largely in urban areas. Processes such as natural plant growth, biomass burning, and combustion also yield volatile organic compounds (VOCs), which have high vapor pressures and can undergo atmospheric oxidation to form secondary organic aerosol (SOA) through gas-to-particle phase partitioning (condensation or nucleation) with subsequent particle-phase (multiphase) chemical reactions (Grieshop et al., 2009).

With around 600 Tg emitted into the atmosphere per year, isoprene (2-methyl-1,3-butadiene, C_5_H_8_) is the most abundant volatile non-methane hydrocarbon (Guenther et al., 2012). The abundance of isoprene is particularly high in the southeastern US due to emissions from broadleaf deciduous tree species (Guenther et al., 2006). Research over the last decade has revealed that isoprene, via hydroxyl radical (OH)- initiated oxidation, is a major source of SOA (Claeys et al., 2004; Edney et al., 2005; Kroll et al., 2005, 2006; Surratt et al., 2006, 2010; Lin et al., 2012, 2013a). In addition, it is known that SOA formation is enhanced by anthropogenic emissions, namely oxides of nitrogen (NO_*x*_) and sulfur dioxide (SO_2_), that are a source of acidic aerosol onto which photochemical oxidation products of isoprene are reactively taken up to yield a variety of SOA products (Edney et al., 2005; Kroll et al., 2006; Surratt et al., 2006, 2007b, 2010; Linetal., 2013b).

Recent work has begun to elucidate some of the critical intermediates of isoprene oxidation that lead to SOA formation through acid-catalyzed heterogeneous chemistry (Kroll et al., 2005; Surratt et al., 2006). Under low-NO_*x*_ conditions, such as in a pristine environment, multiple isomers of isoprene epoxydiols (IEPOX) have been demonstrated to be critical to the formation of isoprene SOA (Paulot et al., 2009). On advection of IEPOX to an urban environment and mixing with anthropogenic emissions of acidic sulfate aerosol, SOA formation is enhanced (Surratt et al., 2006; Lin et al., 2012, 2013b). This pathway has been shown to yield 2-methyltetrols as major SOA constituents of ambient PM_2.5_ (Claeys et al., 2004; Surratt et al., 2010; Lin et al., 2012). Further work has revealed a number of additional IEPOX-derived SOA tracers, including C_5_-alkene triols (Wang et al., 2005; Lin et al., 2012), *cis*- and *trans*-3- methyltetrahydrofuran-3,4-diols (3-MeTHF-3,4-diols) (Lin et al., 2012; Zhang et al., 2012), IEPOX-derived organosul-fates (OSs) (Lin et al., 2012), and IEPOX-derived oligomers (Lin et al., 2014). Some of the IEPOX-derived oligomers have been shown to contribute to aerosol components known as brown carbon that absorb light in the near ultraviolet (UV) and visible ranges (Lin et al., 2014). Under high-NO_*x*_ conditions, such as encountered in an urban environment, isoprene is oxidized to methacrolein (MACR) and SOA formation occurs via the further oxidation of MACR (Kroll et al., 2006; Surratt et al., 2006) to methacryloyl peroxynitrate (MPAN) (Chan et al., 2010; Surratt et al., 2010; Nguyen et al., 2015). It has recently been shown that when MPAN is oxidized by OH it yields at least two SOA precursors, methacrylic acid epoxide (MAE) and hydroxymethyl-methyl-α-lactone (HMML) (Surratt et al., 2006, 2010; Lin et al., 2013a; Nguyen et al., 2015). Whether SOA precursors are formed under high-or low-NO_*x*_ conditions, aerosol acidity is a critical parameter that enhances the reaction kinetics through acid-catalyzed reactive uptake and multiphase chemistry of either IEPOX or MAE (Surratt et al., 2007b, 2010; Lin et al., 2013b). In addition to MACR, other key oxidation products of isoprene, including glycolaldehyde, methylglyoxal, and hydroxyacetone, can undergo multiphase chemistry to yield their respective OS derivatives (Olsen et al., 2011; Schindelka et al., 2013; Shalamzari et al., 2013; Noziere et al., 2015). However, the contribution of isoprene on the glyoxal-, methylglyoxal-, and hydroxyacetone-derived OS mass concentrations in the atmosphere remains unclear since these SOA tracers can also be formed from a wide variety of biogenic and anthropogenic precursors (Galloway et al., 2009; Liao etal., 2015).

Due to the large emissions of isoprene, an SOA yield of even 1 % would contribute significantly to ambient SOA (Carlton et al., 2009; Henze et al., 2009). This conclusion is supported by measurements showing that up to a third of total fine organic aerosol (OA) mass can be attributed to IEPOX-derived SOA tracers in Atlanta, GA (JST), during summer months (Budisulistiorini et al., 2013, 2015). A recent study in Yorkville, GA (YRK), similarly found that IEPOX-derived SOA tracers comprised 12–19% of the fine OA mass (Lin et al., 2013b). Another Southern Oxidant and Aerosol Study (SOAS) site at Centreville, Alabama (CTR), revealed that IEPOX-SOA contributed 18% of total OA mass (Xu et al., 2015). The individual ground sites corroborate recent aircraft-based measurements made in the Studies of Emissions and Atmospheric Composition, Clouds, and Climate Coupling by Regional Surveys (SEAC4RS) aircraft campaign, which estimated an IEPOX-SOA contribution of 32 % to OA mass in the southeastern US (Hu et al., 2015).

It is clear from the field studies discussed above that particle-phase chemistry of isoprene-derived oxidation products plays a large role in atmospheric SOA formation. How-ever, much remains unknown regarding the exact nature of its formation, limiting the ability of models to accurately account for isoprene SOA (Carlton et al., 2010b; Foley et al., 2010). Currently, traditional air quality models in the southeastern US do not incorporate detailed particle-phase chemistry of isoprene oxidation products (IEPOX or MAE/HMML) and generally underpredict isoprene SOA formation (Carlton et al., 2010a). Recent work demonstrates that incorporating the specific chemistry of isoprene epoxide precursors into models increases the accuracy and number of isoprene SOA predictions (Pye et al., 2013; Karambelas et al., 2014; McNeilll, 2015), suggesting that understanding the formation mechanisms of biogenic SOA, especially with regard to the effects of anthropogenic emissions, such as NO_*x*_ and SO_2_, will be key to more accurate models. More accurate models are needed in order to devise cost-effective control strategies for reducing PM_2.5_ levels. Since isoprene is primarily biogenic in origin, and therefore not controllable, the key to understanding the public health and environmental implications of isoprene SOA lies in resolving the effects of anthropogenic pollutants.

This study presents results from the 2013 SOAS, where several well-instrumented ground sites dispersed through-out the southeastern US made intensive gas- and particle-phase measurements from 1 June to 16 July 2013. The primary purpose of this campaign was to examine, in greater detail, the formation mechanisms, composition, and properties of biogenic SOA, including the effects of anthropogenic emissions. This study pertains specifically to the results from the Birmingham, Alabama (BHM), ground site, where the city’s substantial urban emissions mix with biogenic emissions from the surrounding rural areas, creating an ideal location to investigate such interactions. The results presented here focus on analysis of PM_2.5_ collected on filters during the campaign by gas chromatography-electron ionization-mass spectrometry (GC/EI-MS) and ultra performance liquid chromatography coupled to electrospray ion-ization high-resolution quadrupole time-of-flight mass spectrometry (UPLC/ESI-HR-QTOFMS). The analysis of PM_2.5_ was conducted in order to determine quantities of known isoprene SOA tracers using collocated air quality and meteorological measurements to investigate how anthropogenic pollutants including NO_*x*_, SO_2_, aerosol acidity (pH), PM_2.5_ sulfate (${\mathrm{SO}}_{4}^{2-}$), and O_3_ affect isoprene SOA formation. These results, along with the results presented from similar studies during the 2013 SOAS campaign, seek to elucidate the chemical relationships between anthropogenic emissions and isoprene SOA formation in order to provide better parameterizations needed to improve the accuracy of air quality models in this region of the US.

## 2 Methods

### 2.1 Site description and collocated data

Filter samples were collected in the summer of 2013 as part of the SOAS field campaign at the BHM ground site (33.553° N, 86.815° W). In addition to the SOAS campaign, the site is also part of the Southeastern Aerosol Research and Characterization Study (SEARCH) (Fig. S1 of the Supplement), an observation and monitoring program initiated in 1998. SEARCH and this site are described elsewhere in detail (Hansen et al., 2003; Edgerton et al., 2006). The BHM site is surrounded by significant transportation and industrial sources of PM. West of BHM are US 31 and I-65 highways. To the north, northeast and southwest of BHM several coking ovens and an iron pipe foundry are located (Hansen et al., 2003).

### 2.2 High-volume filter sampling and analysis methods

#### 2.2.1 High-volume filter sampling

From 1 June to 16 July 2013, PM_2.5_ samples were collected onto Tissuquartz™ Filters (8 × 10 in., Pall Life Sciences) using high-volume PM_2.5_ samplers (Tisch Environmental) operated at 1 m^3^ min^−1^ at an ambient temperature described in detail elsewhere (Budisulistiorini et al., 2015; Riva et al., 2016). All quartz filters were prebaked prior to collection. The procedure consisted of baking filters at 550 °C for 18 h followed by cooling to 25 °C over 12 h.

The sampling schedule is given in Table 1. Either two or four samples were collected per day. The regular schedule consisted of two samples per day - one during the day, the second at night - each collected for 11 h. On intensive sampling days, four samples were collected, with the single daytime sample being subdivided into three separate periods. The intensive sampling schedule was conducted on days when high levels of isoprene, ${\mathrm{SO}}_{4}^{2-}$ and NO_*x*_ were forecast by the National Center for Atmospheric Research (NCAR) using the Flexible Particle dispersion model (FLEXPART) (Stohl et al., 2005) and Model for Ozone and Related Chemical Tracers (MOZART) (Emmons et al., 2010) simulations. Details of these simulations have been summarized in Bud-isulistiorini et al. (2015); however, these model data were only used qualitatively to determine the sampling schedule. The intensive collection frequency allowed enhanced time resolution for offline analysis to examine the effect of anthro-pogenic emissions on the evolution of isoprene SOA tracers throughout the day.

In total, 120 samples were collected throughout the field campaign with a field blank filter collected every 10 days to identify errors or contamination in sample collection and analysis. All filters were stored at −20 °C in the dark until extraction and analysis. In addition to filter sampling of PM_2.5_, SEARCH provided a suite of additional instruments at the site that measured meteorological and chemical variables, including temperature, relative humidity (RH), solar radiation (SR), barometric pressure (BP), trace gases (i.e., CO, O_3_, SO_2_, NO_*x*_, and NH_3_), and continuous PM monitoring. The exact variables measured with their respective instrumentation are summarized in Table S1 of the Supplement.

#### 2.2.2 Isoprene-derived SOA analysis by GC/EI-MS

SOA collected in the field on quartz filters was extracted and isoprene tracers quantified by GC/EI-MS with prior trimethylsilylation. A 37 mm diameter circular punch from each filter was extracted in a pre-cleaned scintillation vial with 20 mL of high-purity methanol (LC-MS CHROMA-SOLV grade, Sigma-Aldrich) by sonication for 45 min. The extracts were filtered through polytetrafluorethylene (PTFE) syringe filters (Pall Life Science, Acrodisc^®^, 0.2-μm pore size) to remove insoluble particles and residual quartz fibers. The filtrate was then blown dry under a gentle stream of N_2_ at room temperature. The dried residues were immediately trimethylsilylated by reaction with 100 μL of N,O-bis(trimethylsilyl)trifluoroacetamide (BSTFA) + trimethylchlorosilane (TMCS) (99:1 *v*/*v*, Supelco) and 50 μL of pyridine (anhydrous, 99.8%, Sigma-Aldrich) at 70 °C for 1 h. Trimethylsilyl derivatives of carbonyl and hydroxyl functional groups were measurable by our GC/EI-MS method. Derivatized samples were analyzed within 24 h after trimethylsilylation using a Hewlett-Packard (HP) 5890 Series II gas chromatograph coupled to an HP 5971A mass selective detector. The gas chromatograph was equipped with an *Econo-Cap®-EC®−5* Capillary Column (30 m x 0.25 mm i.d.; 0.25 pm film thickness) to separate trimethylsilyl derivatives before MS detection. 1 μL aliquots were injected onto the column. Operating conditions and procedures have been described elsewhere (Surratt et al., 2010).

Extraction efficiency was assessed and taken into account for the quantification of all SOA tracers. Efficiency was determined by analyzing four prebaked filters spiked with 50 ppmv of 2-methyltetrols, 2-methylglyceric acid, levoglucosan, and *cis*- and *trans*-3 —MeTHF-3,4-diols. Extraction efficiency was above 90 % and was used to correct the quantification of samples. Extracted ion chromatograms (EICs) of *m/z* 262, 219, 231, and 335 were used to quantify the cis-/trans-3-MeTHF-3,4-diols, 2-methyltetrols and 2-methylglyceric acid, C_5_-alkene triols, and IEPOX-dimers, respectively (Surratt et al., 2006).

2-Methyltetrols were quantified using an authentic reference standard that consisted of a mixture of racemic dias-teroisomers. Similarly, 3-MeTHF-3,4-diol isomers were also quantified using authentic standards; however, 3-MeTHF-3,4-diol isomers were detected in few field samples. 2-Methylglyceric acid was also quantified using an authentic standard. Procedures for synthesis of the 2-methyltetrols, 3-MeTHF-3,4-diol isomers, and 2-methylglyceric acid have been described elsewhere (Zhang et al., 2012; Budisulistiorini et al., 2015). C_5_-alkene triols and IEPOX dimers were quantified using the average response factor of the 2-methyltetrols.

To investigate the effect of IEPOX-derived OS hydrolysis or decomposition during GC/EI-MS analysis, known concentrations (i.e., 1, 5, 10, and 25 ppmv) of the authentic IEPOX-derived OS standard (Budisulistiorini et al., 2015) were directly injected into the GC/EI-MS following trimethylsilylation. Ratios of detected 2-methyltetrols to the IEPOX-derived OS were applied to estimate the total IEPOX-derived SOA tracers in order to avoid double counting when combining the GC/EI-MS and UPLC/ESI-HR-QTOFMS SOA tracer results.

#### 2.2.3 Isoprene-derived SOA analysis by UPLC/ESI-HR-QTOFMS

A 37 mm diameter circular punch from each quartz filter was extracted following the same procedure as described in Sect. 2.2.2 for the GC/EI-MS analysis. However, after drying, the dried residues were reconstituted with 150 μL of a 50: 50 (*v/v*) solvent mixture of methanol (LC-MS CHROMASOVL grade, Sigma-Aldrich) and high-purity water (Milli-Q, 18.2 ΜΩ). The extracts were immediately analyzed by the UPLC/ESI-HR-QTOFMS (6520 Series, Agilent) operated in the negative ion mode. Detailed operating conditions have been described elsewhere (Riva et al., 2016). Mass spectra were acquired at a mass resolution of 7000–8000.

Extraction efficiency was determined by analyzing three prebaked filters spiked with propyl sulfate and octyl sulfate (electronic grade, City Chemical LLC). Extraction efficiencies were in the range of 86–95%. EICs of *m/z* 215, 333, and 199 were used to quantify the IEPOX-derived OS, IEPOX-derived dimer OS, and the MAE-derived OS, respectively (Surratt et al., 2007a). EICs were generated with a ±5 ppm tolerance. Accurate masses for all measured organosulfates (OSs) were within ±5 ppm. For simplicity, only the nominal masses are reported in the text when describing these products. IEPOX-derived OS and IEPOX-derived dimer OS were quantified by the IEPOX-derived standard synthesized in-house (Budisulistiorini et al., 2015). The MAE-derived OS was quantified using an authentic MAE-derived OS standard synthesized in-house by a procedure to be described in a forthcoming publication (^1^H nuclear magnetic resonance (NMR) trace, Fig. S2). Although the MAE-derived OS (Gomez-Gonzalez et al., 2008), which is more formally called 3-sulfooxy-2-hydroxy-2-methyl propanoic acid, has been chemically verified from the reactive uptake of MAE on wet acidic sulfate aerosol (Lin et al., 2013a), the term MAE/HMML-derived OS will be used hereafter to denote the two potential precursors (MAE and HMML) contributing to this OS derivative as recently discussed by Nguyen et al. (2015). It should be noted that Nguyen et al. (2015) provided indirect evidence for the possible existence of HMML. As a result, further work is needed to synthesize this compound to confirm its structure and likely role in SOA formation from isoprene oxidation.

EICs of *m/z* 155, 169, and 139 were used to quantify the glyoxal-derived OS, methylglyoxal-derived OS, and the hydroxyacetone-derived OS, respectively (Surratt et al., 2007a). In addition, EICs of *m/z* 211, 260, and 305 were used to quantify other known isoprene-derived OSs (Surratt et al., 2007a). Glycolic acid sulfate synthesized in-house was used as a standard to quantify the glyoxal-derived OS (Galloway et al., 2009), and propyl sulfate was used as a surrogate standard to quantify the remaining isoprene-derived OSs.

#### 2.2.4 OC and WSOC analysis

A 1.5 cm^2^ square punch from each quartz filter was analyzed for total organic carbon (OC) and elemental carbon (EC) by the thermal-optical method (Birch and Cary, 1996) on a Sunset Laboratory OC-EC instrument (Tigard, OR) at the National Exposure Research Laboratory (NERL) at the US Environmental Protection Agency, Research Triangle Park, NC. The details of the instrument and analytical method have been described elsewhere (Birch and Cary, 1996). In addition to the internal calibration using methane gas, four different mass concentrations of sucrose solution were used to verify the accuracy of the instrument during the analysis.

Water-soluble organic carbon (WSOC) was measured in aqueous extracts of quartz fiber filter samples using a total organic carbon (TOC) analyzer (Sievers 5310C, GE Water & Power) equipped with an inorganic carbon remover (Sievers 900). To maintain low background carbon levels, all glass-ware used was washed with water, soaked in 10 % nitric acid, and baked at 500 °C for 5 h and 30 min prior to use. Samples were extracted in batches that consisted of 12–21 PM_2.5_ samples and field blanks, one laboratory blank, and one spiked solution. A 17.3 cm^2^ filter portion was extracted with 15 mL of purified water (> 18Ω Barnstead Easypure II, Thermo Scientific) by ultra-sonication (Branson 5510). Extracts were then passed through a 0.45 μm PTFE filter to remove insoluble particles. The TOC analyzer was calibrated using potassium hydrogen phthalate (KHP, Sigma-Aldrich) and was verified daily with sucrose (Sigma-Aldrich). Samples and standards were analyzed in triplicate; the reported values correspond to the average of the second and third trials. Spiked solutions yielded recoveries that averaged (±1 standard deviation) 96 ± 5 % (*n* = 9). All ambient concentrations were field blank subtracted.

#### 2.2.5 Estimation of aerosol pH by ISORROPIA

Aerosol pH was estimated using a thermodynamic model, ISORROPIA-II (Nenes et al., 1998). ${\mathrm{SO}}_{4}^{2-}$, nitrate (${\mathrm{NO}}_{3}^{-}$), and ammonium (${\mathrm{NH}}_{4}^{+}$) ion concentrations measured in PM_2.5_ collected from BHM, as well as RH, temperature, and gas-phase ammonia (NH3) were used as inputs into the model. These variables were obtained from the SEARCH network at BHM, which collected the data during the period covered by the SOAS campaign. The ISORROPIA-II model estimates particle hydronium ion concentration per unit volume of air (H^+^, μg m^−3^), aerosol liquid water content (LWC, μgm^−3^), and aqueous aerosol mass concentration (μgm^−3^). The model-estimated parameters were used in the following formula to calculate the aerosol pH:

(1) $$\mathrm{Aerosol}\;pH=-\log_{10}a_{H^{+}}=-\log_{10}\left(\frac{H_{\mathrm{air}}^{+}}{\mathrm{LMASS}}\times \rho_{\mathrm{aer}}\times 1000\right),$$

where $a_{H^{+}}$ is H^+^ activity in the aqueous phase (molL^−1^), LMASS is total liquid-phase aerosol mass (μgm^−3^), and *ρ*_er_ is aerosol density. Details of the ISORROPIA-II model and its ability to predict pH, LWC, and gas-to-particle partitioning are not the focus of this study and are discussed elsewhere (Fountoukis et al., 2009).

#### 2.2.6 Estimation of nighttime NO_3_

Nitrate radical (NO_3_) production (P[NO_3_]) was calculated using the following equation:

(2) $$P[{\mathrm{NO}}_{3}]=k[{\mathrm{NO}}_{2}][O_{3}],$$

where [NO_2_] and [O_3_] correspond to the measured ambient NO_2_ and O_3_ concentrations (mol cm^−3^), respectively, and *k* is the temperature-dependent rate constant (Herron and Huie, 1974; Graham and Johnston, 1978). Since no direct measure of NO_3_ radical was made at this site during SOAS, P[NO_3_] was used as a proxy for NO_3_ radicals present in the atmo-sphere to examine whether there is any association of it with isoprene-derived SOA tracers.

## 3 Results and discussion

### 3.1 Overview of the study

**Figure 1.**
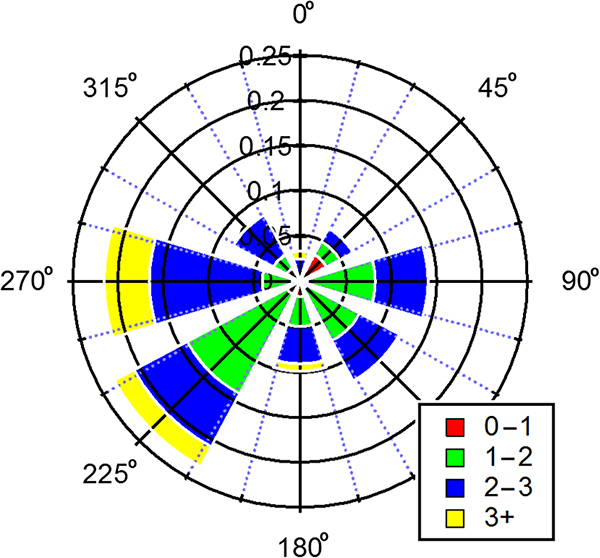
Wind rose illustrating wind direction during the campaign at the BHM site. Bars indicate direction of incoming wind, with 0° set to geographic north. Length of bar size indicates frequency with color segments indicating the wind speed in meters per second.

**Figure 2.**
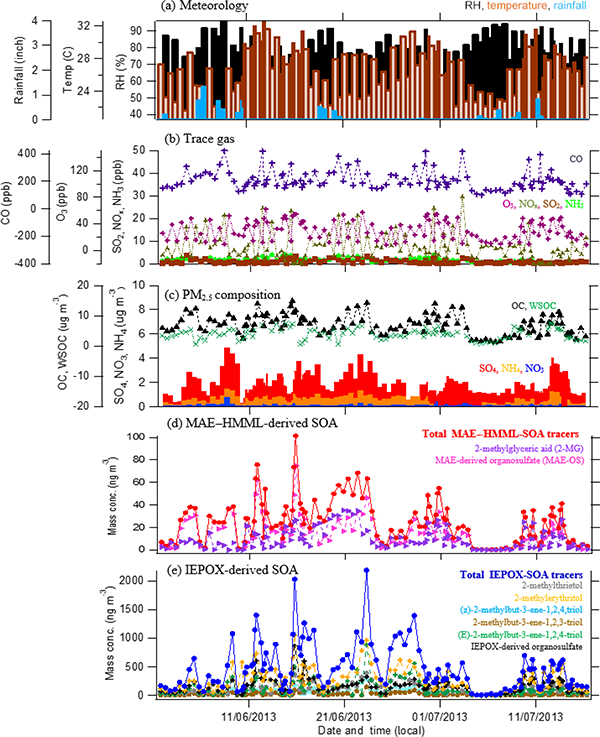
Time series of **(a)** meteorological data, **(b)** trace gases, **(c)** PM_2.5_ constituents, **(d)** MAE/HMML-derived SOA tracers, and **(e)** IEPOX-derived SOA tracers during the 2013 SOAS campaign at the BHM site.

The campaign extended from 1 June through 16 July 2013. Temperature during this period ranged from a high of 32.6 ° C to a low of 20.5 °C, with an average of 26.4 °C. RH varied from 37–96 % throughout the campaign, with an average of 71.5%. Rainfall occurred intermittently over 2–3-day periods and averaged 0.1 in. per day. Wind analysis reveals that air masses approached largely from the south-southeast at an average wind speed of 2 ms^-1^. Summaries of meteorological conditions as well as wind speed and direction during the course of the campaign are given in Table 2 and illustrated in Figs. 1 and 2.

The average concentration of carbon monoxide (CO), a combustion byproduct, was 208.7 ppbv. The mean concentration of O_3_ was significantly higher (*t* test, *p* value <0.05) on intensive sampling days (37.0 ppbv) compared to regular sampling days (25.2ppbv). Campaign average concentrations of NO_*x*_, NH_3_, and SO_2_ were 7.8, 1.9, and 0.9ppbv, respectively. On average, OC and WSOC levels were 7.2 (*n* = 120) and 4 μgm^−3^ (*n* = 100), respectively. The largest inorganic component of PM_2.5_ was ${\mathrm{SO}}_{4}^{2-}$, which averaged 2μgm^−3^ with excursions between 0.4 and 4.9 μgm^−3^ during the campaign. ${\mathrm{NH}}_{4}^{+}$and ${\mathrm{NO}}_{3}^{-}$ were present at low levels, averaging 0.66 and 0.14 μgm^−3^, respectively. Time series of gas and PM_2.5_ components are shown in Fig. 2. WSOC accounted for 35% of OC mass (Fig. S3a), and was smaller than that recently reported in rural areas during SOAS (Bud-isulistiorini et al., 2015; Hu et al., 2015) but consistent with previous observations at the BHM site (Ding et al., 2008). WSOC / OC ratios are commonly lower in urban than rural areas, as a consequence of higher primary OC emissions; thus, PM at BHM probably contains increased OC.

Diurnal variation of meteorological parameters, trace gases, and PM_2.5_ components is shown in Fig. S4 of the Supplement. Temperature dropped during nighttime and reached a maximum in the afternoon (Fig. S4a). Conversely, RH was low during the day and high at night. High NO_*x*_ levels were found in the early morning and decreased during the course of the day (Fig. S4c), most likely due to the formation of NO_*x*_ sinks (e.g., RONO_2_, ROONO_2_, and HNO_3_) as well as possibly due to increasing planetary boundary layer (PBL) heights. O_3_ reached a maximum concentration between 12:00–15:00 local time due to photochemistry (Fig. S4b). SO_2_ was slightly higher in the morning (Fig. S4c) but decreased during the day, most likely as a result of PBL dynamics. NH_3_ remained fairly constant throughout the day (Fig. S4c). No significant diurnal variation was found in the concentration of inorganic PM_2.5_ components, including ${\mathrm{SO}}_{4}^{2-}$, ${\mathrm{NO}}_{3}^{-}$, and ${\mathrm{NH}}_{4}^{+}$ (Fig. S4d). Unfortunately, a measurement of isoprene could not be made at BHM during the campaign. However, the diurnal trend of isoprene levels might be similar to the data at the CTR site (Xu et al., 2015), which is only 61 miles away from BHM. Xu et al. (2015) observed the highest levels of isoprene (~ 6 ppb) at CTR in the midafternoon (15:00 local time) and its diurnal trend was similar to isoprene OA measured by the Aerodyne Aerosol Mass Spectrometer (AMS) during the SOAS campaign at the CTR site.

### 3.2 Characterization of isoprene SOA

Table 3 summarizes the mean and maximum concentrations of known isoprene-derived SOA tracers detected by GC/EI-MS and UPLC/ESI-HR-QTOFMS. Levoglucosan was also analyzed as a tracer for biomass burning. Among the isoprene-derived SOA tracers, the highest mean concentration was for 2-methyltetrols (376 ngm^−3^), followed by the sum of C_5_-alkene triols (181 ngm^−3^) and the IEPOX-derived OS (165ngm^−3^). The concentrations account for 3.8, 1.8, and 1.6%, respectively, of total OM mass. It is noteworthy that maximum concentrations of 2-methylerythritol (a 2-methyltetrol isomer; 1049ngm^−3^), IEPOX-derived OS (865 ng m^−3^), and (E)-2-methylbut-3-ene-1,2,4-triol (879ngm^−3^) were attained during the intensive sampling period of 16:00–19:00 local time on 15 June 2013, following 5 consecutive days of dry weather (Fig. 2a and d) when high levels of isoprene, ${\mathrm{SO}}_{4}^{2-}$, and NO_*x*_ were forecast.

Our investigation for the potential of OS hydrolysis or decomposition during GC/EI-MS analysis demonstrated that only 1.7% of 2-methylthreitol and 2.4% of 2-methylerythritol could be derived from the IEPOX-derived OSs. In order to accurately estimate the mass concentrations of the IEPOX-derived SOA tracers, we took this effect into account. Together, the IEPOX-derived SOA tracers, which represent SOA formation from isoprene oxidation predominantly under the low-NO_*x*_ pathway, comprised 92.45 % of the total detected isoprene-derived SOA tracer mass at the BHM site. This contribution is slightly lower than observations reported at rural sites located in Yorkville, GA (97.50 %), and Look Rock, Tennessee (LRK) (97 %) (Lin et al., 2013b; Budisulistiorini et al., 2015).

The sum of MAE/HMML-OS and 2-methylglyceric acid (2-MG), which represent SOA formation from isoprene oxidation predominantly under the high-NOx pathway, contributed 3.25 % of the total isoprene-derived SOA tracer mass, while the OS derivative of glycolic acid (GA sulfate) contributed 3.3 %. The contribution of GA sulfate was consistent with the level of GA sulfate measured by the airborne NOAA Particle Analysis Laser Mass Spectrometer (PALMS) over the continental US during the Deep Convective Clouds and Chemistry Experiment and SEAC4RS (Liao et al., 2015). However, the contribution of GA sulfate to the total OM at BHM (0.3 %) is lower than aircraft-based measurements made by Liao et al. (2015) near the ground in the eastern US (0.9%). GA sulfate can form from biogenic and anthropogenic emissions other than isoprene, including glyoxal, which is thought to be a primary source of GA sulfate (Galloway et al., 2009). For this reason, GA sulfate will not be further discussed in this study.

Isoprene SOA contribution to total OM was estimated by assuming the OM / OC ratio of 1.6 based on recent studies (El-Zanan et al., 2009; Simon et al., 2011; Ruthenburg et al., 2014; Blanchard et al., 2016). On average, isoprene-derived SOA tracers (sum of both IEPOX- and MAE/HMML-derived SOA tracers) contributed ~ 7 % (ranging up to ~ 20 % at times) of the total particulate OM mass. The average contribution is lower than that measured at other sites in the SE USA, including both rural LRK (Budisulistiorini et al., 2015; Hu et al., 2015) and urban Atlanta, GA (Budisulistiorini et al., 2013). The contribution of SOA tracers to OM in the current study was estimated on the basis of the offline analysis of filters, while tracer estimates in the two earlier studies were based on online Aerodyne Aerosol Chemical Speciation Monitor (ACSM)/AMS measurements. The low isoprene SOA / OM ratio is consistent with the low WSOC / OC ratio reported in Sect. 3.1, suggesting a larger contribution of primary OA or hydrophobic secondary OM originating from anthropogenic emissions to the total OM at BHM. However, it should be noted that total IEPOX-derived SOA mass at BHM may actually be closer to ~ 14% since recent measurements by the Aerodyne ACSM at LRK indicated that tracers could only account for ~ 50 % of the total IEPOX-derived SOA mass resolved by the ACSM (Budisulistiorini et al., 2015). Unfortunately, an Aerodyne ACSM or AMS was not available at the BHM site to confirm that IEPOX-derived SOA mass at BHM might account for 14 % (on average) of the total OM mass.

Levoglucosan, a biomass-burning tracer, averaged 1 % of total OM with spikes up to 8 %, the same level measured for 2-methylthreitol and (E)-2-methylbut-3-ene-1,2,4-triol (Table 3). The ratio of average levoglucosan at BHM relative to CTR was 5.4, suggesting significantly more biomass burning impacting the BHM site.

IEPOX- and MAE/HMML-derived SOA tracers accounted for 18 and 0.4% of the WSOC mass, respectively (Fig. S3b), lower than the respective contributions of 24 and 0.7 % measured at LRK (Budisulistiorini et al., 2015).

**Figure 3.**
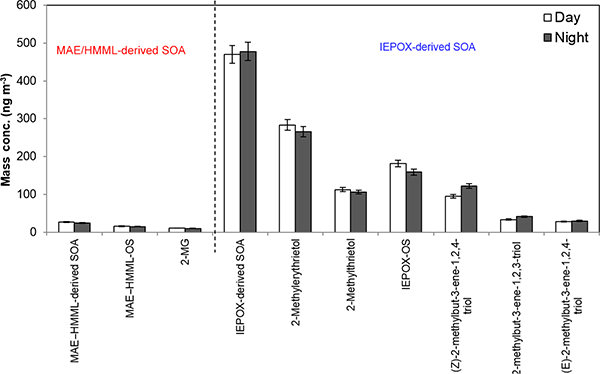
The bar chart shows average daytime and nighttime concentrations of isoprene-derived SOA tracers with 95 % confident interval. No significant variation between daytime and nighttime was observed.

**Figure 4.**
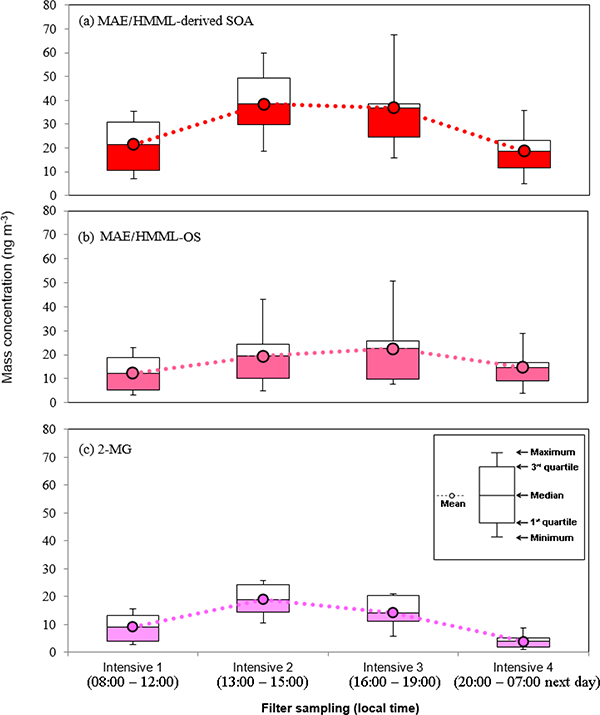
The box-and-whisker plot (n = 15) of **(a)** MAE/HMML-derived SOA, **(b)** MAE/HMML-OS, and **(c)** 2-MG. These demonstrate that the statistical distribution of SOA abundance during each intensive sampling period. No significant variation amongst intensive samples was observed.

**Figure 5.**
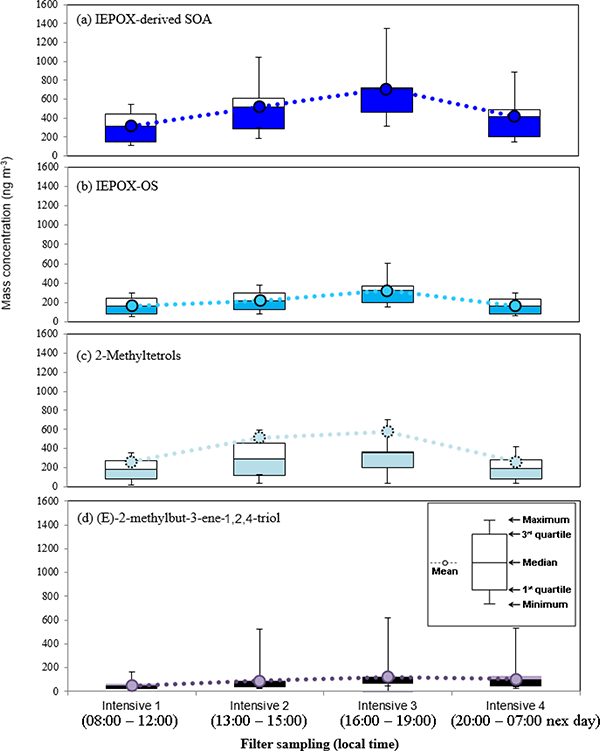
The box-and-whisker plot (n = 15) of **(a)** IEPOX-derived SOA, **(b)** IEPOX-OS, **(c)** 2-methyltetrols, and **(d)** (E)-2-methylbut-3-ene-1,2,4-triol. These demonstrate that the statistical distribution of SOA abundance during each intensive sampling period. No significant variation amongst intensive samples was observed.

Figure 3 shows no difference for the average day and night concentration of isoprene-derived SOA tracers, suggesting that the majority of isoprene SOA tracers are potentially long-lived and formed upwind. A recent study by Lopez-Hilfiker et al. (2016) at the CTR site during the 2013 SOAS demonstrated that isoprene-derived SOA was comprised of effectively nonvolatile material, which could allow for this type of SOA to be long-lived in the atmosphere. Although 2-MG and MAE-derived OS are known to form under high-NO_*x*_ conditions (Lin et al., 2013a), no correlation between 2-MG and MAE-derived OS with NO_*x*_ (Table 4) is observed at the BHM. This supports the idea that isoprene SOA tracers likely formed at upwind locations and were subsequently transported to the sampling site. Higher isoprene emissions during the daytime and cooler nighttime temperatures do not appear to cause any differences between daytime and nighttime isoprene-derived SOA tracer concentrations. Figures 4 and 5 show the variation of isoprene-derived SOA tracers during intensive sampling periods. The highest concentrations were usually observed in samples collected from 16:00 to 19:00, local time; however, no statistically significant difference was observed between intensive periods. This observation illustrates the importance of the higher time resolution of the tracer data during intensive sampling periods over the course of the campaign (Tables S2-S6). An additional consequence of the intensive sampling periods was the resolution of a significant correlation between isoprene SOA tracers and O_3_, to be discussed in more detail in Sect. 3.3.2.

### 3.3 Influence of anthropogenic emissions on isoprene-derived SOA

#### 3.3.1 Effects of reactive nitrogen-containing species

**Figure 6.**
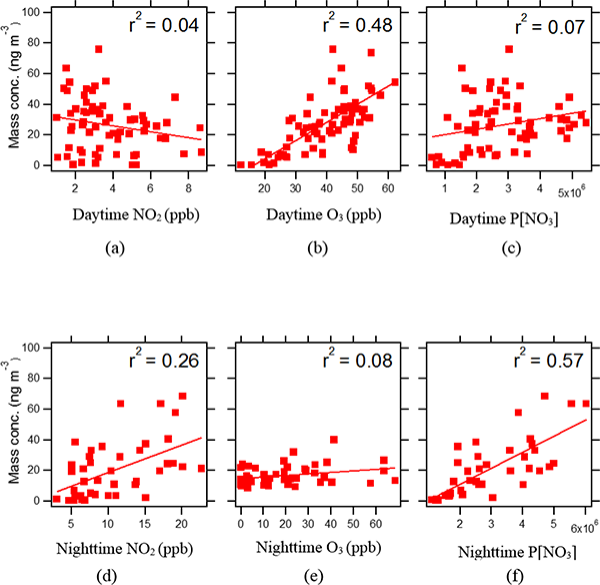
Correlation of MAE/HMML-derived SOA tracers with **(a)** daytime NO_2_, **(b)** daytime O_3_, **(c)** daytime P[NO_3_], **(d)** night-time NO_2_, **(e)** nighttime O_3_, and **(f)** nighttime P[NO_3_]. Nighttime P[NO_3_] correlation suggests that NO_3_ radical chemistry could explain some fraction of the MAE/HMML-derived SOA tracer concentrations.

**Figure 7.**
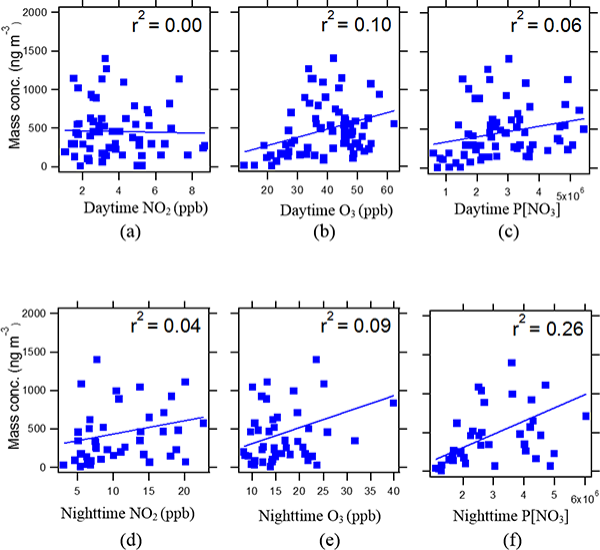
Correlation of IEPOX-derived SOA tracers with **(a)** daytime NO_2_, **(b)** daytime O_3_, **(c)** daytime P[NO_3_], **(d)** nighttime NO_2_, **(e)** nighttime O_3_, and **(f)** nighttime P[NO_3_]. Nighttime P[NO_3_] correlation suggests that NO_3_ radical chemistry could explain some fraction ofthe IEPOX-derived SOA tracer concentrations. The contribution of nighttime P[NO_3_] to IEPOX-derived SOA would be smaller than MAE/HMML-derived SOA due to the weaker correlation.

During the campaign, no isoprene-derived SOA tracers, including MAE/HMML-derived OS and 2-MG, correlated with NO_*x*_ or NO_*y*_ (*r*^2^ = 0, *n* = 120). This is inconsistent with the current understanding of SOA formation from isoprene oxidation pathways under high-NO_*x*_ conditions, which proceeds through the uptake of MAE (Lin et al., 2013a), and, as recently suggested, HMML (Nguyen et al., 2015), to yield 2-MG and its OS derivative. Plume age, as a ratio of NO_*x*_ :NO_*y*_, in this study was highly correlated with O_3_ (*r*^2^ = 0.79, *n* = 120), which is consistent with the relative diurnal variation of NO_*x*_, NO_*y*_, and O_3_ as discussed in Sect. 3.1. This correlation might also be explained by the photolysis of NO_2_, which is abundant due to traffic at the urban ground site, resulting in the formation of tropospheric O_3_. A negative correlation coefficient (*r*^2^ = 0.22, *n* = 120) between plume age and 2-MG abundance was found as a consequence of relative diurnal variations. The peak of 2-MG was observed in the afternoon after NO_*x*_ has decreased. This correlation leads to the hypothesis that the formation of 2-MG may be associated with the ageing of air masses; however, further investigation is warranted. A previous study supported a major role for NO_3_ in the nighttime chemistry of isoprene (Ng et al., 2008). Correlation of IEPOX- and MAE/HMML-derived SOA with nighttime NO_2_, O_3_, and P[NO_3_] were examined in this study (Figs. 6 and 7). As shown in Fig. 6f, a moderate correlation between MAE/HMML-derived SOA and night-time P[NO_3_] (*r*^2^ = 0.57, *n* = 40) was observed. The regression analysis revealed a significant correlation at the 95 % confidence interval (*p* value <0.05) (Table S7). This finding suggests that some MAE/HMML-derived SOA may form locally from the reaction of isoprene with the NO_3_ radical at night. A field study reported a peak isoprene mixing ratio in the early evening (Starn et al., 1998) as the PBL height decreases at night. As a result, lowering PBL heights could concentrate the remaining isoprene, NO_2_, and O_3_ that can continue to react during the course of the evening. 2-MG formation has been reported to be NO_2_-dependent via the formation and further oxidation of MPAN (Surratt et al., 2006; Chan et al., 2010). Hence, decreasing PBL may be related to nighttime MAE/HMML-derived SOA formation through isoprene oxidation by both P[NO_3_] and NO_2_.

Although P[NO_3_] depends on both NO_2_ and O_3_ levels, O_3_ correlates moderately with MAE/HMML-derived SOA tracers during the day (*r*^2^ = 0.48, *n* = 75) but not at night (*r*^2^ = 0.08, *n* = 45). The effect of O_3_ on isoprene-derived SOA formation during daytime will be discussed further in Sect. 3.3.2. NO_2_ levels correlate only weakly with MAE/HMML-derived SOA tracers (*r*^2^ = 0.26, *n* = 45), indicating that NO2 levels alone do not explain the moderate correlation of P[NO_3_] with these tracers. To our knowledge, the correlation of P[NO_3_] with high-NO_*x*_ SOA tracers has not been observed in previous field studies, indicating that further work is needed to examine the potential role of night-time NO_3_ radicals in forming these SOA tracers.

As shown in Fig. 7f, IEPOX-derived SOA was weakly correlated (*r*^2^ = 0.26, *n* = 40) with nighttime P[NO_3_]. The correlation appears to be driven by the data at the low end of the scale and could therefore be misleading. However, Schwantes et al. (2015) demonstrated that NO_3_-initiated oxidation of isoprene yields isoprene nitrooxy hydroperoxides (INEs) through the nighttime reaction of RO_2_ + HO_2_, which upon further oxidation yielded isoprene nitrooxy hydroxye-poxides (INHEs). The INHEs undergo reactive uptake onto acidic sulfate aerosol to yield SOA constituents similar to those of IEPOX-derived SOA. The present study raises the possibility that a fraction of IEPOX-derived SOA comes from NO_3_-initiated oxidation of isoprene at night. The work of Ng et al. (2008), which only observed SOA as a consequence of the RO_2_ + RO_2_ and RO_2_ + NO_3_ reactions dominating the fate of the RO2 radicals, does not explain the weak association between IEPOX-derived SOA tracers and P[NO_3_] which we observe in this study. It is now thought that RO_2_ + HO_2_ should dominate the fate of RO_2_ radicals in the atmosphere (Paulot et al., 2009; Schwantes et al., 2015).

#### 3.3.2 Effect of O_3_

During the daytime, O_3_ was moderately correlated (*r*^2^ = 0.48, *n* = 75) with total MAE/HMML-derived SOA (Fig. 6b). This correlation was stronger (*r*^2^ = 0.72, *n* = 30, p value <0.05; Table S7) when filters taken during regular daytime sampling periods are considered, suggesting that the formation of MACR (a precursor to MAE and HMML) (Lin et al., 2013b; Nguyen et al., 2015) was enhanced by oxidation of isoprene by O_3_ (Kamens et al., 1982). O_3_ was not correlated (*r*^2^ = 0.08, *n* = 45) with MAE/HMML-derived SOA at night (Fig. 6e). The latter finding is consistent with the absence of photolysis to drive the production of O_3_. However, residual O_3_ may play an important role at night to form MAE/HMML-derived SOA via the P[NO_3_] pathway discussed in Sect. 3.3.1.

O_3_ was not correlated (*r*^2^ = 0.10, *n* = 75) with IEPOX-derived SOA during daytime (Fig. 7b) but weakly correlated with 2-methylerythritol (*r*^2^ = 0.25, *n* = 30) as shown in Table S2, especially during Intensive 3 sampling periods (*r*^2^ = 0.34, *n* = 15; Table S5). An important observation with regard to this result is that no correlation has been found between O_3_ and 2-methyltetrols (*r*^2^<0.01) in previous field studies (Lin et al., 2013b; Budisulistiorini et al., 2015). Isoprene ozonolysis yielded 2-methyltetrols in chamber studies in the presence of acidified sulfate aerosol (Riva et al., 2016), but C_5_-alkene-triols were not formed by this pathway. The greatest abundance of isoprene-derived SOA tracers in day-time samples was generally observed in Intensive 3 samples; however, there was no statistically significant difference observed between intensive samples. The moderate correlation (*r*^2^ = 0.34, *n* = 15, p value <0.05) between O_3_ and the 2-methyltetrols observed in Intensive 3 samples occurred when O_3_ reached maximum levels, suggesting that ozonolysis of isoprene plays a role in 2-methyltetrol formation. Lack of correlation between O_3_ and C_5_-alkene triols during Intensive 3 sampling (*r*^2^ = 0.10, *n* = 15) supports this contention. Previous studies (Nguyen et al., 2010; Inomata et al., 2014) proposed that SOA formation from isoprene ozonolysis occurs from stabilized Criegee intermediates (sCIs) that can further react in the gas phase to form higher molecular weight products that subsequently partition to the aerosol phase to make SOA. Recent work by Riva et al. (2016) systematically demonstrated that isoprene ozonolysis in the presence of wet acidic aerosol yields 2-methyltetrols and organosul-fates unique to this process. Notably, no C_5_-alkene triols were observed, which are known to form simultaneously with 2-methyltetrols if IEPOX multiphase chemistry is involved (Lin et al., 2012). Riva et al. (2016) tentatively proposed that hydroperoxides formed in the gas phase from isoprene ozonolysis potentially partition to wet acidic sulfate aerosols and hydrolyze to yield 2-methyltetrols as well as the unique set of organosulfates observed (Riva et al., 2016). Additional work using authentic hydroperoxide standards is needed to validate this tentative hypothesis.

#### 3.3.3 Effect of particle $SO_{4}^{2–}$

${\mathrm{SO}}_{4}^{2-}$ was moderately correlated with IEPOX-derived SOA (*r*^2^ = 0.36, *n* = 117) and MAE/HMML-derived SOA (*r*^2^ = 0.33, *n* = 117) at the 95% confidence interval as shown in Table S7. The strength of the correlations was consistent with studies at other sites across the southeastern US (Bud-isulistiorini et al., 2013, 2015; Lin et al., 2013b; Xu et al., 2015). Aerosol surface area provided by acidic ${\mathrm{SO}}_{4}^{2-}$ has been demonstrated to control the uptake of isoprene-derived epoxides (Lin et al., 2012; Gaston et al., 2014; Nguyen et al., 2014; Riedel et al.,2015).

Furthermore, ${\mathrm{SO}}_{4}^{2-}$ is proposed to enhance IEPOX-derived SOA formation by providing particle water (H2O_ptcl_) required for IEPOX uptake (Xu et al., 2015). Aerosol ${\mathrm{SO}}_{4}^{2-}$ also promotes acid-catalyzed ring-opening reactions of IEPOX by H^+^, proton donors such as${\mathrm{NH}}_{4}^{+}$, and nucleophiles (e.g., H_2_O, ${\mathrm{SO}}_{4}^{2-}$, or${\mathrm{NO}}_{3}^{-}$) (Surratt et al., 2010; Nguyen et al., 2014). Since ${\mathrm{SO}}_{4}^{2-}$ tends to drive both particle water and acidity (Fountoukis and Nenes, 2007), the extent to which each influences isoprene SOA formation during field studies remains unclear. Multivariate linear regression analysis on SOAS data from the CTR site and the Southeastern Center for Air Pollution and Epidemiology (SCAPE) data set revealed a statistically significant positive linear relationship between ${\mathrm{SO}}_{4}^{2-}$ and the isoprene (IEPOX) OA factor resolved by positive matrix factorization (PMF). On the basis of this analysis the abundance of ${\mathrm{SO}}_{4}^{2-}$ was concluded to control the isoprene SOA formation over broad areas of the southeastern US directly (Xu et al., 2015), consistent with previous reports (Lin et al., 2013; Budisulistiorini et al., 2013, 2015). Another potential pathway for ${\mathrm{SO}}_{4}^{2-}$ levels to enhance isoprene SOA formation is through salting-in effects, through which the solubility of polar organic compounds would be increased in aqueous solution with increasing salt concentration (Xu et al., 2015). However, systematic investigations of this effect are lacking and further studies are warranted.

#### 3.3.4 Effect of aerosol acidity

The aerosol at BHM was acidic throughout the SOAS campaign (pH range 1.60–1.94, average 1.76) in accordance with a study by Guo et al. (2015) that found aerosol pH ranging from 0 to 2 throughout the southeastern US. However, no correlation of pH with isoprene SOA formation was observed at BHM, also consistent with previous findings using the thermodynamic models to estimate aerosol acidity in many field sites across the southeastern US region, including YRK (Lin et al., 2013b), JST (Budisulistiorini et al., 2013), and LRK (Budisulistiorini et al., 2015). However, it is important to point out that the lack of correlation between SOA tracers and acidity may stem from the small variations in aerosol acidity and the fact that aerosols are very acidic throughout the campaign. Gaston et al. (2014) and Riedel et al. (2015) recently demonstrated that an aerosol pH < 2 at atmospherically relevant aerosol surface areas would allow the reactive uptake of IEPOX onto acidic (wet) sulfate aerosol surfaces to be competitive with other loss processes (e.g., deposition and reaction of IEPOX with OH). In fact, it was estimated that under such conditions IEPOX would have a lifetime of ~ 5 h. The constant presence of acidic aerosol has also been observed at other field sites in the southeastern US (Bud-isulistiorini et al., 2013,^2015^; Xu et al., 2015), supporting the conclusion that acidity is not the limiting variable in forming isoprene SOA.

### 3.4 Comparison among different sampling sites during 2013 SOAS campaign

Table 5 summarizes the mean concentration and contribution of each isoprene SOA tracer at BHM, CTR, and LRK. BHM is an industrial-residential area; LRK and CTR are rural areas, although LRK is influenced by a diurnal upslope-downslope cycle of air from an urban locality (Knoxville) (Tanner et al., 2005). IEPOX-derived SOA (isoprene SOA produced under low-NO_*x*_ conditions) was predominant at all three sites during the SOAS campaign, while MAE/HMML-derived SOA (isoprene SOA produced under high-NO_*x*_ conditions) constituted a minor contribution. The average ratio of 2-methyltetrols to C_5_-alkene triols at BHM was 2.2, nearly double that of CTR (1.3) and LRK (1.1). Although 2-methyltetrols and C_5_-alkene triols are considered to form readily from the acid-catalyzed reactive uptake and multiphase chemistry of IEPOX (Edney et al., 2005; Surratt et al., 2006), Riva et al. (2016) recently demonstrated that only 2-methyltetrols can be formed via isoprene ozonolysis in the presence of acidic sulfate aerosol. The detailed mechanism explaining isoprene ozonolysis is still unclear, but acid-catalyzed heterogeneous reaction with organic peroxides or H2 O2 was considered to be a possible route for 2-methyltetrol formation. The higher levels of the 2-methyltetrols observed at the urban BHM site indicate a likely competition between the IEPOX uptake and ozonolysis pathways. Together, these findings suggest that urban O_3_ may play an important role in forming the 2-methyltetrols observed at BHM. There were notable trends found among the three sites: (1) average C_5_-alkene triol concentrations were higher at CTR (214.1 ngm^−3^) than at BHM (169.7ngm^−3^) and LRK (144.4ngm^−3^); (2) average isomeric 3-MeTHF-diol concentrations were lower at CTR (0.2ngm^−3^) than the BHM (15.4ngm^−3^) or LRK (4.4 ng m^−3^) sites. Except for the 2-methyltetrols, reasons for the differences observed for the other tracers between sites remain unclear and warrant future investigations.

## 4 Conclusions

This study examined isoprene SOA tracers in PM_2.5_ samples collected at the BHM ground site during the 2013 SOAS campaign and revealed the complexity and potential multitude of chemical pathways leading to isoprene SOA formation. Isoprene SOA contributed up to ~ 20 % (~ 7 % on average) of total OM mass. IEPOX-derived SOA tracers were responsible for 92.45 % of the total quantified isoprene SOA tracer mass, with 2-methyltetrols being the major component (47 %). Differences in the relative contributions of IEPOX- and MAE/HMML-derived SOA tracers at BHM and the rural CTR and LRK sites (Budisulistiorini et al., 2015) during the 2013 SOAS campaign support suggestions that anthropogenic emissions affect isoprene SOA formation. The correlation between 2-methyltetrols and O_3_ at BHM is in accordance with work by Riva et al. (2016), demonstrating a potential role of O_3_ in generating isoprene-derived SOA in addition to the currently accepted IEPOX multiphase pathway.

At BHM, the statistical correlation of particulate ${\mathrm{SO}}_{4}^{2-}$ with IEPOX-(*r*^2^ = 0.36, *n* = 117, p<0.05) and MAE-derived SOA tracers (*r*^2^ = 0.33, *n* = 117, p<0.05) suggests that ${\mathrm{SO}}_{4}^{2-}$ plays a role in isoprene SOA formation. Although none of the isoprene-derived SOA tracers correlated with gas-phase NO_*x*_ and NO_*y*_, MAE/HMML-derived SOA tracers correlated with nighttime P[NO_3_] (*r*^2^ = 0.57, *n* = 40), indicating that NO_3_ may affect local MAE/HMML-derived SOA formation. Nighttime P[NO_3_] was weakly correlated (*r*^2^ = 0.26, *n* = 40) with IEPOX-derived SOA tracers, lending some support to recent work by Schwantes et al. (2015) showing that isoprene + NO_3_ yields INHEs that can undergo reactive uptake to yield IEPOX tracers and contribute to IEPOX-derived SOA tracer loadings. The correlation of daytime O_3_ with MAE/HMML-derived SOA and with 2-methyltetrols offers a new insight into influences on isoprene SOA formation. Notably, O_3_ has not been reported to cor-relate with isoprene-derived SOA tracers in previous field studies (Lin et al., 2013b; Budisulistiorini et al., 2015). In this study, the strong correlation (*r*^2^ = 0.72, *n* = 30) at the 95 % confidence interval of O_3_ with MAE/HMML-derived SOA tracers during the regular daytime sampling schedule indicates that O_3_ likely oxidizes some isoprene to MACR as a precursor of 2-MG at BHM. The weak correlation (*r*^2^ = 0.16, *n* = 75) between O_3_ and 2-methyltetrols early in the day as well as the better correlation (*r*^2^ = 0.34, *n* = 15) later in the day (Intensive 3, 16:00–19:00 local time) are consistent with recent laboratory studies demonstrating that 2-methyltetrols can be formed via isoprene ozonolysis in the presence of acidified sulfate aerosol (Riva et al., 2016).

Although urban O_3_ and nighttime P[NO_3_] may have a role in local formation of MAE/HMML- and IEPOX-derived SOA tracers at BHM, this does not appear to explain the majority of the SOA tracers, since no significant day-night variation of the entire group of tracers was observed during the campaign. The majority of IEPOX-derived SOA was likely formed when isoprene SOA precursors (IEPOX) were generated upwind and transported to the BHM site. Wind directions during the campaign are consistent with long-range transport of isoprene SOA precursors from southwest of the site, which is covered by forested areas. The absence of a correlation of aerosol acidity with MAE/HMML- and IEPOX-derived SOA tracers indicates that acidity is not the limiting variable that controls the formation of these compounds. Because the aerosols are acidic (campaign average aerosol pH of 1.8), the lack of correlation between SOA tracers and acidity may stem from the nearly invariant aerosol acidity throughout the campaign. Hence, despite laboratory studies demonstrating that aerosol acidity can enhance isoprene SOA formation (Surratt et al., 2007, 2010; Lin et al., 2012), the effect may not be significant in the southeastern US during the summer months due to the constant acidity of aerosols. Future work should examine how well current models can predict the isoprene SOA levels observed during this study, especially in the presence of fresh urban emissions. Further-more, explicit models are now available to predict the isoprene SOA tracers measured here (McNeill et al., 2012; Pye et al., 2013), which will allow the modeling community to test the current parameterizations that are used to capture the enhancing effect of anthropogenic pollutants on isoprene-derived SOA formation. In addition, the significant correlations of isoprene-derived SOA tracers with P[NO_3_] observed during this study indicate a need to better understand night-time chemistry of isoprene. Lastly, although O_3_ appears to have an enhancing effect on isoprene-derived SOA tracers, the intermediates are unknown. Hydroperoxides suggested by Riva et al. (2016) may be key, but chamber experiments with authentic precursors are needed to test this hypothesis.

## Supplementary Material

Supp

## Figures and Tables

**Table 1.** Sampling schedule during SOAS at the BHM ground site.

No. of samples per day	Sampling schedule	Dates
Two (regular)	Day: 08:00–19:00	1–9 June
	Night: 20:00–07:00 next day	13 June
		17–28 June
		2–9 July
		15 July

Four (intensive)	Intensive 1: 08:00–12:00	10–12 June
	Intensive 2: 13:00–15:00	14–16 June
	Intensive 3: 16:00–19:00	29–30 June
	Intensive 4: 20:00–07:00 next day	1,9–14 July

**Table 2.** Summary of collocated measurements of meteorological variables, gaseous species, and PM_2.5_ constituents.

Category	Condition	Average	SD	Minimum	Maximum
Meteorology	Rainfall (in.)	0.1	0.2	0.0	1.4
	Temp (°C)	26.4	3.0	20.5	32.7
	RH (%)	71.5	15.0	36.9	96.1
	BP (mbar)	994.2	3.9	984.2	1002.4
	SR (W m^−2^)	303.7	274.5	7.0	885.0

Trace gas (ppbv)	O_3_	31.1	14.8	8.3	62.2
	CO	208.7	72.0	99.6	422.9
	SO_2_	0.9	0.8	0.1	3.7
	NO	1.3	1.2	0.1	7.0
	NO_2_	6.6	5.1	1.0	22.7
	NO_*x*_	7.8	6.0	1.3	29.7
	NO_*y*_	9.1	5.8	2.2	30.4
	HNO_3_	0.3	0.2	0.1	1.0
	NH_3_	1.9	0.8	0.7	4.0

PM_2.5_ (μgm^−3^)	OC	7.2	3.2	1.4	14.9
	EC	0.6	0.5	0.1	2.7
	WSOC	4.0	1.8	0.5	7.5
	${\mathrm{SO}}_{4}^{2-}$	2.0	0.9	0.4	4.9
	${\mathrm{NO}}_{3}^{-}$	0.1	0.1	0.0	0.8
	${\mathrm{NH}}_{4}^{+}$	0.7	0.3	0.2	1.2
	Aerosol pH	1.8	0.1	1.6	1.9

**Table 3.** Summary of isoprene-derived SOA tracers measured by GC/EI-MS and UPLC/ESI-HR-QTOFMS.

SOA tracers	*m/z*	Frequency of detection (%)^a^	Max concentration (ng m^−3^)	Mean concentration (ng m^−3^)	Isoprene SOA mass fraction (%)^b^	% of total OM^c^
Measured by GC/EI-MS						

2-methylerythritol^d^	219	99.2	1048.9	269.0	33.8	2.7
2-methylthreitol^d^	219	100.0	388.9	107.3	13.5	1.1
(E)-2-methylbut-3-ene-1,2,4-triol^e^	231	96.7	878.9	112.7	14.2	1.1
(Z)-2-methylbut-3-ene-1,2,4-triol^e^	231	95.8	287.8	38.9	4.9	0.4
2-methylbut-3-ene-1,2,3-triol^e^	231	94.2	503.3	28.9	3.6	0.3
2-methylglyceric acid^d^	219	93.3	35.0	10.8	1.4	0.1
*cis*-3-MeTHF-3,4-diol^d^	262	22.5	98.9	6.9	0.9	0.1
*trans*-3-MeTHF-3,4-diol^d^	262	10.0	137.6	8.6	1.1	0.1
IEPOX-derived dimer^e^	333	10.0	2.2	0.0	0.0	0.0
Levoglucosan^d^	204	100.0	922.6	98.7	-	1.0

Measured by UPLC/DAD-ESI-HR-QTOFMS						

IEPOX-derived OSs						
C_5_H_11_O_7_S^−d^	215	100.0	864.9	164.5	20.7	1.6
C_10_H_21_O_10_S^−f^	333	1.7	0.3	0.0	0.0	0.0
MAE-derived OS^d^						
C_4_H_7_O_7_S^−^	199	100.0	35.7	7.2	1.9	0.1
GA sulfate^d^						
C_2_H_3_O_6_S^−^	155	100.0	75.2	26.2	3.3	0.3
Methylglyoxal-derived OS^g^						
C_3_H_5_O_6_S^−^	169	97.5	10.5	2.7	0.3	0.0
Isoprene-derived OSs^g^						
C_5_H_7_O_7_S^−^	211	97.5	5.2	1.4	0.2	0.0
C_5_h_10_no_9_S^−^	260	90.0	3.9	0.3	0.0	0.0
C_5_H_9_N_2_O_11_S^−^	305	5.0	3.3	2.9	0.4	0.0
Hydroxyacetone-derived OS^g^						
C_2_H_3_O_5_S^−^	139	30.8	2.6	0.2	0.0	0.0

a Total filters: 120.

b Mass fraction is the contribution of each species among total known isoprene-derived SOA mass detected by GC/EI-MS and UPLC/DAD-ESI-HR-QTOFMS.

c OM / OC = 1.6.

d OA tracers quantified by authentic standards.

e SOA tracers quantified by 2-methyltetrols as a surrogate standard.

f SOA tracer quantified by IEPOX-derived OS (*m/z* 215) as a surrogate standard.

g SOA tracers quantified by propyl sulfate as a surrogate standard.

**Table 4.** Overall correlation (*r*^2^) of isoprene-derived SOA tracers and collocated measurements at BHM during 2013 SOAS campaign.

SOA tracers	CO	O_3_	NO*x*	NO*y*	SO_2_	NH_3_	SO_4_	NO_3_	NH_4_	OC	WSOC	pH
MAE/HMML-derived SOA tracers^*^	0.07	0.26	0.00	0.01	0.06	0.11	0.33	0.01	0.18	0.47	0.20	0.00

2-Methylglyceric acid	0.01	0.26	0.01	0.00	0.01	0.07	0.10	0.00	0.06	0.19	0.02	0.00
MAE-derived OS	0.10	0.14	0.00	0.02	0.07	0.09	0.38	0.01	0.18	0.32	0.23	0.01

IEPOX-derived SOA tracers^**^	0.04	0.05	0.00	0.01	0.05	0.01	0.36	0.00	0.21	0.24	0.12	0.00

2-Methylerythritol	0.00	0.16	0.03	0.02	0.01	0.00	0.30	0.02	0.18	0.18	0.19	0.00
2-Methylthreitol	0.00	0.13	0.02	0.03	0.02	0.00	0.20	0.01	0.16	0.17	0.15	0.00
(E)-2-methylbut-3-ene-1,2,4-triol	0.07	0.00	0.02	0.01	0.07	0.00	0.15	0.00	0.19	0.11	0.04	0.00
(Z)-2-methylbut-3-ene-1,2,4-triol	0.04	0.00	0.00	0.00	0.06	0.00	0.28	0.00	0.20	0.04	0.00	0.00
2-methylbut-3-ene-1,2,3-triol	0.02	0.00	0.03	0.00	0.00	0.02	0.32	0.01	0.03	0.17	0.04	0.00
IEPOX-derived OS	0.02	0.14	0.03	0.00	0.00	0.00	0.27	0.00	0.16	0.29	0.29	0.00
IEPOX dimer	0.00	0.00	0.00	0.00	0.00	0.00	0.00	0.00	0.00	0.00	0.00	0.00

Other isoprene SOA tracers												

GA sulfate C_2_H_3_O_6_S^-^	0.30	0.23	0.01	0.00	0.08	0.09	0.27	0.00	0.19	0.38	0.18	0.00
Methylglyoxal-derived OS												
C_3_H_5_O_6_S^-^ Isoprene-derived OSs	0.14	0.04	0.02	0.03	0.03	0.07	0.31	0.02	0.25	0.21	0.24	0.00
C_5_H_7_O_7_S^-^	0.01	0.23	0.03	0.01	0.00	0.02	0.21	0.00	0.16	0.31	013	0.00
C_5_HioNO_9_S^-^	0.17	0.00	0.12	0.14	0.10	0.14	0.31	0.16	0.23	0.20	0.07	0.00
C_5_H_9_N_2_O_11_S^-***^	0.32	0.71	0.66	0.58	0.42	0.02	0.68	0.50	0.42	0.00	0.50	0.00
Hydroxyacetone-derived OS												
C_2_H_3_O_5_S^-^	0.02	0.10	0.08	0.07	0.05	0.00	0.00	0.03	0.00	0.01	0.01	0.00

Other tracer												

Levoglucosan	0.00	0.09	0.02	0.01	0.02	0.00	0.00	0.02	0.00	0.08	0.04	0.01

* Summed tracers for MAE/HMML-derived SOA.

** Summed tracers for IEPOX-derived SOA.

*** Found only in 6 of 120 filters.

The correlations in this table are positive.

**Table 5.** Summary of isoprene-derived SOA tracers from the three SOAS ground sites: BHM, CTR, and LRK.

SOA tracers	Urban		Rural			
	
	BHM		CTR		LRK	
	
	Mean (ngm^−3^)	Average fraction of detected tracers (%)	Mean (ngm^−3^ )	Average fraction of detected tracers (%)	Mean (ngm^−3^)	Average fraction of detected tracers (%)
MAE/HMML-derived SOA						

MAE/HMML-derived OS	7.2	1.1	10.2	1.3	8.2	1.8
2-Methylglyceric acid	10.4	1.7	5.1	0.7	7.5	1.6

IEPOX-derived SOA						

IEPOX-derived OS	164.5	24.3	207.1	26.8	139.2	30.3
IEPOX-derived dimer OS	0.04	0.00	0.7	0.1	1.1	0.2
2-Methylerythritol	266.7	37.9	204.8	26.5	120.7	26.3
2-Methylthreitol	107.3	15.8	73.7	9.5	42.4	9.2
(E)-2-Methylbut-3-ene-1,2,4- triol	109.0	12.3	137.3	17.8	98.8	21.5
(Z)-2-Methylbut-3-ene-1,2,4- triol	37.3	4.1	50.7	6.6	29.1	6.1
2-Methylbut-3-ene-1,2,3-triol	23.4	2.5	26.1	3.4	16.5	3.6
*trans*-3-MeTHF-3,4-diol	8.6	1.0	0.0	0.0	2.7	0.6
*cis*-3-MeTHF-3,4-diol	6.8	1.0	0.2	0.0	1.7	0.4
