# Supplementary material for: Assessing the impact of anthropogenic pollution on isoprene-derived secondary organic aerosol formation in PM2.5 collected from the Birmingham, Alabama, ground site during the 2013 Southern Oxidant and Aerosol Study
Source: Atmos Chem Phys. Author manuscript; Available in PMC 2018 Sep 19. (PMC6145830; doi:10.5194/acp-16-4897-2016)
Supplement: Supp [file NIHMS982682-supplement-Supp.pdf]

Supplement of Atmos. Chem. Phys., 16, 4897–4914, 2016
http://www.atmos-chem-phys.net/16/4897/2016/
doi:10.5194/acp-16-4897-2016-supplement
© Author(s) 2016. CC Attribution 3.0 License.

Atmospheric Chemistry and Physics
Open Access

Supplement of

Assessing the impact of anthropogenic pollution on isoprene-derived secondary organic aerosol formation in $PM_{2.5}$ collected from the Birmingham, Alabama, ground site during the 2013 Southern Oxidant and Aerosol Study

Weruka Rattanavaraha et al.

Correspondence to: J. D. Surratt (surratt@unc.edu)

The copyright of individual parts of the supplement might differ from the CC-BY 3.0 licence.

Table S1. Instrumentation and time resolution of collocated measurements at BHM.

**Category**	**Variable**	**Analyzer/Sensor**	**Time Resolution (Interval, average) (minutes)**
**Meteorology**	Wind Speed/Direction	RMYoung 81000 sonic	5, 60
	T/RH/BP	Paroscientific Met4A	5, 60
	T/RH	Vaisala	5, 60
	PAR	Licor	5, 60
	Precipitation	ETI-NOAH IV	5, 60
	Aerosol/cloud layers	JenOptik CHM 15k ceilometer	5, 60
	Surface wetness	Vaisala (SWS2)	5, 60
**Trace Gases**	O_3	Thermo 49i	5, 60
	CO	Thermo 48i	5, 60
	SO_2	Thermo 43i	5, 60
	NO	Thermo 42i	5, 60
	NO_2	Photolysis/Thermo 49i	5, 60
	HNO_3	Continuous denuder diff/Thermo 42i	5, 60
	NO_y	Cat. reduction/Thermo 42i	5, 60
	NH_3	Continuous denuder diff/Thermo 42i	5, 60
**Continuous PM**	$PM_{2.5}$ Mass	TEOM	60
	PMcoarse Mass	Dichotomous TEOM	60
	$PM_{2.5}$ SO_4	Cat. reduction/Thermo 43i	60
	$PM_{2.5}$ NO_3	Cat. reduction/Thermo 42i	60
	$PM_{2.5}$ NH_4	Cat. oxidation/Thermo 42i	60
	$PM_{2.5}$ TC/EC	Sunset	60
	Dry Babs (880 nm)	Radiance Research M903	5, 60
	Dry Bsp (530 nm)	Magee 2ch. Aeth	5, 60
	Ambient Bsp (530 nm)	Optec NGN-2a	5, 60
**Filter-Based PM**	$PM_{2.5}$ Mass	gravimetry	1440, daily
	$PM_{2.5}$ ions	IC	1440, 1 in 3 days
	$PM_{2.5}$ major/minor elements	XRF	1440, daily
	$PM_{2.5}$ water-soluble metals	ICPMS	1440, 1 in 3 days
	$PM_{2.5}$ OC/EC	TOR	1440, 1 in 3 days
	PM_{coarse} Mass	gravimetry	1440, 1 in 3 days
	PM_{coarse} ions	IC	1440, 1 in 3 days
	PM_{coarse} major/minor elements	XRF	1440, 1 in 3 days
	PM_{coarse} water-soluble metals	ICPMS	1440, 1 in 3 days
**Hi-Vol Based PM**	$PM_{2.5}$ OC/EC	TOR	23-hr, daily
	$PM_{2.5}$ ions	IC	23-hr, daily
	$PM_{2.5}$ (other)	Various	11-hr, daily

Table S2. Correlation (r^2) of isoprene-derived SOA tracers and collocated measurements during regular day sampling (8 am – 7 pm).

SOA tracers	CO	O_3	NO_x	NO_y	SO_2	NH_3	SO_4	NO_3	NH_4	OC	WSOC	pH
**MAE/HMML-derived SOA tracers**	**0.31**	**0.72**	**0.04**	**0.00**	**0.20**	**0.34**	**0.51**	**0.10**	**0.53**	**0.44**	**0.48**	**0.01**
2-methylglyceric acid	0.14	0.44	0.01	0.00	0.09	0.15	0.19	0.03	0.27	0.09	0.12	0.00
MAE-derived OS	0.28	0.60	0.04	0.00	0.14	0.31	0.66	0.14	0.56	0.58	0.52	0.01
**IEPOX-derived SOA tracers**	**0.09**	**0.26**	**0.01**	**0.01**	**0.08**	**0.12**	**0.41**	**0.04**	**0.41**	**0.31**	**0.32**	**0.01**
2-methylerythritol	0.04	0.30	0.03	0.00	0.05	0.04	0.31	0.00	0.31	0.24	0.30	0.01
2-methylthreitol	0.02	0.20	0.02	0.00	0.06	0.03	0.21	0.00	0.23	0.13	0.19	0.00
(E)-2-methylbut-3-ene-1,2,4-triol	0.05	0.24	0.02	0.00	0.03	0.05	0.33	0.02	0.32	0.22	0.27	0.00
(Z)-2-methylbut-3-ene-1,2,4-triol	0.10	0.11	0.00	0.01	0.09	0.17	0.34	0.10	0.32	0.24	0.16	0.01
2-methylbut-3-ene-1,2,3-triol	0.11	0.11	0.00	0.01	0.09	0.18	0.36	0.10	0.34	0.25	0.17	0.01
IEPOX-derived OS	0.17	0.41	0.01	0.01	0.08	0.19	0.47	0.07	0.50	0.53	0.59	0.01
IEPOX dimer	0.00	0.00	0.00	0.00	0.00	0.00	0.00	0.00	0.00	0.00	0.00	0.00
**Other isoprene SOA tracers**												
GA sulfate												
$C_2H_3O_6S^-$	0.22	0.20	0.00	0.00	0.07	0.19	0.49	0.20	0.39	0.33	0.21	0.01
Methylglyoxal-derived OS												
$C_3H_5O_6S^-$	0.25	0.40	0.01	0.01	0.11	0.11	0.57	0.05	0.46	0.41	0.47	0.01
Isoprene-derived OSs												
$C_5H_7O_7S^-$	0.13	0.34	0.01	0.01	0.02	0.17	0.35	0.11	0.40	0.21	0.28	0.00
$C_5H_{10}NO_9S^-$	0.02	0.37	0.12	0.06	0.00	0.01	0.48	0.12	0.38	0.18	0.12	0.11
$C_5H_9N_2O_{11}S^-$ *	0.25	0.56	0.48	0.40	0.15	0.40	0.52	0.28	0.24	0.57	0.46	0.00
Hydroxyacetone-derived OS												
$C_2H_3O_5S^-$	0.42	0.73	0.06	0.16	0.00	0.18	0.55	0.23	0.71	0.57	0.66	0.00
**Other tracer**												
Levoglucosan	0.26	0.34	0.00	0.00	0.09	0.21	0.44	0.10	0.47	0.22	0.25	0.01

* Found only in 6 of 120 filters

The correlations in this table are positive.

Table S3. Correlation (r^2) of isoprene-derived SOA tracers and collocated measurements during intensive 1 sampling (8 am – 11 am).

SOA tracers	CO	O_3	NO_x	NO_y	SO_2	NH_3	SO_4	NO_3	NH_4	OC	WSOC	pH
**MAE/HMML-derived SOA tracers**	**0.00**	**0.20**	**0.04**	**0.16**	**0.01**	**0.07**	**0.35**	**0.25**	**0.46**	**0.47**	**0.16**	**0.18**
2-methylglyceric acid	0.03	0.22	0.05	0.10	0.00	0.07	0.00	0.43	0.11	0.46	0.07	0.08
MAE-derived OS	0.01	0.09	0.02	0.12	0.01	0.03	0.72	0.06	0.62	0.26	0.08	0.18
**IEPOX-derived SOA tracers**	**0.11**	**0.04**	**0.05**	**0.00**	**0.06**	**0.26**	**0.30**	**0.00**	**0.16**	**0.04**	**0.02**	**0.03**
2-methylerythritol	0.15	0.01	0.02	0.00	0.16	0.52	0.22	0.03	0.18	0.00	0.00	0.15
2-methylthreitol	0.04	0.00	0.00	0.00	0.10	0.19	0.13	0.02	0.16	0.00	0.02	0.13
(E)-2-methylbut-3-ene-1,2,4-triol	0.12	0.03	0.06	0.01	0.01	0.27	0.23	0.00	0.09	0.11	0.05	0.00
(Z)-2-methylbut-3-ene-1,2,4-triol	0.13	0.02	0.05	0.01	0.03	0.32	0.28	0.00	0.08	0.09	0.05	0.00
2-methylbut-3-ene-1,2,3-triol	0.07	0.02	0.02	0.00	0.02	0.26	0.22	0.01	0.03	0.04	0.28	0.01
IEPOX-derived OS	0.09	0.07	0.07	0.00	0.05	0.19	0.30	0.00	0.17	0.04	0.00	0.02
IEPOX dimer	0.00	0.00	0.00	0.00	0.00	0.00	0.00	0.00	0.00	0.00	0.00	0.00
**Other isoprene SOA tracers**												
GA sulfate												
$C_2H_3O_6S^-$	0.00	0.19	0.03	0.01	0.01	0.03	0.37	0.02	0.44	0.25	0.11	0.00
Methylglyoxal-derived OS												
$C_3H_5O_6S^-$	0.05	0.05	0.18	0.28	0.02	0.00	0.01	0.11	0.24	0.09	0.56	0.03
Isoprene-derived OSs												
$C_5H_7O_7S^-$	0.09	0.15	0.00	0.20	0.05	0.02	0.36	0.12	0.25	0.40	0.00	0.02
$C_5H_{10}NO_9S^-$	0.00	0.05	0.02	0.06	0.06	0.04	0.38	0.00	0.23	0.17	0.18	0.37
$C_5H_9N_2O_{11}S^-$ *	0.00	0.00	0.00	0.00	0.00	0.21	0.00	0.00	0.00	0.00	0.00	0.00
Hydroxyacetone-derived OS												
$C_2H_3O_5S^-$	0.25	0.67	0.71	0.65	0.21	0.21	0.03	0.26	0.12	0.50	0.00	0.70
**Other tracer**												
Levoglucosan	0.03	0.07	0.02	0.00	0.07	0.07	0.08	0.11	0.01	0.03	0.02	0.24

* Found only in 6 of 120 filters
The correlations in this table are positive.

Table S4. Correlation (r^2) of isoprene-derived SOA tracers and collocated measurements during intensive 2 sampling (12 pm – 3 pm).

SOA tracers	CO	O_3	NO_x	NO_y	SO_2	NH_3	SO_4	NO_3	NH_4	OC	WSOC	pH
**MAE/HMML-derived SOA tracers**	**0.13**	**0.42**	**0.0**	**0.12**	**0.04**	**0.01**	**0.14**	**0.05**	**0.29**	**0.55**	**0.19**	**0.00**
2-methylglyceric acid	0.01	0.47	0.25	0.32	0.00	0.04	0.00	0.05	0.04	0.17	0.07	005
MAE-derived OS	0.15	0.20	0.04	0.01	0.06	0.00	0.18	0.15	0.31	0.49	0.24	0.03
**IEPOX-derived SOA tracers**	**0.22**	**0.00**	**0.04**	**0.08**	**0.00**	**0.21**	**0.34**	**0.32**	**0.37**	**0.46**	**0.81**	**0.02**
2-methylerythritol	0.41	0.00	0.13	0.14	0.01	0.16	0.48	0.24	0.50	0.42	0.77	0.01
2-methylthreitol	0.29	0.00	0.03	0.07	0.00	0.07	0.22	0.41	0.39	0.32	0.70	0.02
(E)-2-methylbut-3-ene-1,2,4-triol	0.17	0.00	0.04	0.07	0.01	0.17	0.30	0.31	0.29	0.44	0.61	0.02
(Z)-2-methylbut-3-ene-1,2,4-triol	0.21	0.00	0.05	0.07	0.01	0.17	0.33	0.29	0.31	0.45	0.64	0.01
2-methylbut-3-ene-1,2,3-triol	0.03	0.02	0.00	0.02	0.03	0.07	0.13	0.21	0.06	0.09	0.62	0.03
IEPOX-derived OS	0.19	0.02	0.11	0.21	0.00	0.32	0.43	0.16	0.39	0.52	0.58	0.00
IEPOX dimer	0.00	0.00	0.00	0.00	0.00	0.00	0.00	0.00	0.00	0.00	0.00	0.00
**Other isoprene SOA tracers**												
GA sulfate												
$C_2H_3O_6S^-$	0.24	0.23	0.00	0.08	0.00	0.06	0.32	0.23	0.46	0.46	0.48	0.00
Methylglyoxal-derived OS												
$C_3H_5O_6S^-$	0.27	0.28	0.01	0.02	0.01	0.06	0.29	0.00	0.29	0.33	0.43	0.03
Isoprene-derived OSs												
$C_5H_7O_7S^-$	0.14	0.02	0.06	0.07	0.03	0.06	0.16	0.00	0.18	0.18	0.09	0.00
$C_5H_{10}NO_9S^-$	0.00	0.15	0.07	0.05	0.21	0.34	0.03	0.05	0.00	0.06	0.00	0.18
$C_5H_9N_2O_{11}S^{-}$ *	0.00	0.00	0.00	0.00	0.00	0.00	0.00	0.00	0.00	0.00	0.00	0.00
Hydroxyacetone-derived OS												
$C_2H_3O_5S^-$	0.09	0.40	0.01	0.01	0.10	0.05	0.04	0.07	0.10	0.07	0.62	0.01
**Other tracer**												
Levoglucosan	0.03	0.00	0.22	0.13	0.00	0.01	0.03	0.17	0.00	0.02	0.00	0.07

* Found only in 6 of 120 filters

The correlations in this table are positive.

Table S5. Correlation (r^2) of isoprene-derived SOA tracers and collocated measurements during intensive 3 sampling (4 pm – 7 pm).

SOA tracers	CO	O_3	NO_x	NO_y	SO_2	NH_3	SO_4	NO_3	NH_4	OC	WSOC	pH
**MAE/HMML-derived SOA tracers**	**0.01**	**0.47**	**0.45**	**0.39**	**0.47**	**0.00**	**0.19**	**0.10**	**0.12**	**0.54**	**0.23**	**0.15**
2-methylglyceric acid	0.12	0.37	0.03	0.17	0.25	0.00	0.00	0.05	0.02	0.34	0.50	0.15
MAE-derived OS	0.00	0.37	0.44	0.39	0.41	0.01	0.25	0.09	0.13	0.45	0.04	0.10
**IEPOX-derived SOA tracers**	**0.10**	**0.15**	**0.18**	**0.14**	**0.50**	**0.17**	**0.47**	**0.00**	**0.18**	**0.31**	**0.24**	**0.03**
2-methylerythritol	0.03	0.34	0.08	0.04	0.58	0.12	0.34	0.01	0.14	0.42	0.22	0.00
2-methylthreitol	0.04	0.32	0.03	0.01	0.43	0.17	0.25	0.03	0.14	0.54	0.21	0.01
(E)-2-methylbut-3-ene-1,2,4-triol	0.00	0.21	0.05	0.02	0.70	0.13	0.33	0.00	0.12	0.38	0.01	0.02
(Z)-2-methylbut-3-ene-1,2,4-triol	0.00	0.21	0.09	0.05	0.77	0.14	0.41	0.00	0.13	0.27	0.01	0.01
2-methylbut-3-ene-1,2,3-triol	0.54	0.00	0.12	0.13	0.00	0.01	0.18	0.04	0.06	0.00	0.33	0.02
IEPOX-derived OS	0.15	0.10	0.17	0.12	0.42	0.16	0.41	0.00	0.15	0.24	0.29	0.03
IEPOX dimer	0.00	0.00	0.00	0.00	0.00	0.00	0.00	0.00	0.00	0.00	0.00	0.00
**Other isoprene SOA tracers**												
GA sulfate												
$C_2H_3O_6S^-$	0.20	0.28	0.43	0.32	0.02	0.00	0.19	0.16	0.30	0.55	0.01	0.21
Methylglyoxal-derived OS												
$C_3H_5O_6S^-$	0.26	0.16	0.01	0.01	0.10	0.12	0.57	0.34	0.60	0.03	0.00	0.02
Isoprene-derived OSs												
$C_5H_7O_7S^-$	0.06	0.18	0.19	0.13	0.12	0.14	0.45	0.02	0.35	0.55	0.02	0.00
$C_5H_{10}NO_9S^-$	0.06	0.45	0.00	0.03	0.80	0.05	0.44	0.03	0.18	0.27	0.15	0.00
$C_5H_9N_2O_{11}S^{-}$ *	0.00	0.00	0.00	0.00	0.00	0.00	0.00	0.00	0.00	0.00	0.00	0.00
Hydroxyacetone-derived OS												
$C_2H_3O_5S^-$	0.49	0.01	0.10	0.20	0.13	0.05	0.44	0.24	0.11	0.06	0.29	0.10
**Other tracer**												
Levoglucosan	0.00	0.01	0.02	0.04	0.00	0.06	0.00	0.02	0.00	0.20	0.01	0.04

* Found only in 6 of 120 filters

The correlations in this table are positive.

Table S6. Correlation (r^2) of isoprene-derived SOA tracers and collocated measurements during intensive 4 and regular nighttime (8 pm – 7 am next day).

SOA tracers	CO	O_3	NO_x	NO_y	SO_2	NH_3	SO_4	NO_3	NH_4	OC	WSOC	pH
**MAE/HMML-derived SOA tracers**	**0.35**	**0.08**	**0.18**	**0.21**	**0.17**	**0.39**	**0.48**	**0.15**	**0.42**	**0.53**	**0.15**	**0.01**
2-methylglyceric acid	0.18	0.00	0.13	0.10	0.12	0.18	0.17	0.05	0.22	0.17	0.01	0.04
MAE-derived OS	0.35	0.14	0.15	0.17	0.11	0.32	0.51	0.17	0.36	0.58	0.20	0.00
**IEPOX-derived SOA tracers**	**0.10**	**0.10**	**0.02**	**0.03**	**0.08**	**0.10**	**0.37**	**0.02**	**0.30**	**0.27**	**0.15**	**0.00**
2-methylerythritol	0.02	0.12	0.00	0.00	0.05	0.01	0.23	0.00	0.20	0.14	0.09	0.00
2-methylthreitol	0.06	0.09	0.00	0.01	0.09	0.05	0.38	0.01	0.30	0.21	0.15	0.00
(E)-2-methylbut-3-ene-1,2,4-triol	0.09	0.08	0.02	0.03	0.10	0.10	0.35	0.03	0.28	0.26	0.11	0.00
(Z)-2-methylbut-3-ene-1,2,4-triol	0.07	0.07	0.01	0.02	0.10	0.07	0.32	0.02	0.27	0.22	0.08	0.00
2-methylbut-3-ene-1,2,3-triol	0.02	0.05	0.00	0.01	0.03	0.02	0.18	0.01	0.16	0.15	0.09	0.00
IEPOX-derived OS	0.17	0.10	0.08	0.10	0.01	0.16	0.27	0.03	0.21	0.31	0.14	0.01
IEPOX dimer	0.00	0.00	0.00	0.00	0.00	0.00	0.00	0.00	0.00	0.00	0.00	0.00
**Other isoprene SOA tracers**												
GA sulfate												
$C_2H_3O_6S^-$	0.12	0.22	0.02	0.04	0.04	0.14	0.28	0.01	0.15	0.31	0.26	0.01
Methylglyoxal-derived OS												
$C_3H_5O_6S^-$	0.16	0.05	0.03	0.05	0.00	0.18	0.19	0.01	0.17	0.26	0.24	0.00
Isoprene-derived OSs												
$C_5H_7O_7S^-$	0.12	0.15	0.01	0.02	0.02	0.09	0.22	0.01	0.11	0.17	0.12	0.00
$C_5H_{10}NO_9S^-$	0.20	0.00	0.11	0.12	0.08	0.21	0.39	0.18	0.33	0.30	0.09	0.00
$C_5H_9N_2O_{11}S^{-}$ *	0.00	0.00	0.00	0.00	0.00	0.00	0.00	0.00	0.00	0.00	0.00	0.00
Hydroxyacetone-derived OS												
$C_2H_3O_5S^-$	0.89	0.09	0.83	0.89	0.30	0.83	0.00	0.00	0.17	0.40	0.59	0.01
**Other tracer**												
Levoglucosan	0.23	0.00	0.22	0.24	0.08	0.21	0.13	0.01	0.18	0.25	0.11	0.00

* Found only in 6 of 120 filters

The correlations in this table are positive.

Table S7. Regression and correlation (r^2) analysis at the 95% confidence interval

	Variables		Regression Statistics					*p-value*
	Y	x	Number of observations	Multiple r	r^2	Adjusted r^2	Standard error	
Nighttime: MAE/HMML-derived SOA vs P[NO_3]	MAE/HMML -derived SOA	P[NO_3]	40	0.7532	0.5673	0.5559	12.5098	2.05E-08
Nighttime: IEPOX-derived SOA vs P[NO_3]	IEPOX-derived SOA	P[NO_3]	40	0.5086	0.2587	0.2392	393.7399	8.05E-04
Regular day sampling: MAE/HMML-derived SOA vs O_3	MAE/HMML -derived SOA	O_3	30	0.8457	0.7153	0.7051	8.9517	4.00E-09
Daytime: 2-methyltetrols vs O_3	2-methyltetrols	O_3	64	0.3610	0.1303	0.1163	254.4175	3.39E-03
Intensive 3: MAE/HMML-derived SOA vs O_3	MAE/HMML -derived SOA	O_3	15	0.6844	0.4683	0.4274	18.3128	4.89E-03
Intensive 3: 2-methyltetrols vs O_3	2-methyltetrols	O_3	15	0.5844	0.3415	0.2908	259.0249	2.22E-02
MAE/HMML-derived SOA vs SO_4	MAE/HMML -derived SOA	SO_4	117	0.5779	0.3340	0.3282	15.8648	8.96E-12
IEPOX-derived SOA vs SO_4	IEPOX-derived SOA	SO_4	117	0.6027	0.3632	0.3577	310.4400	6.51E-13

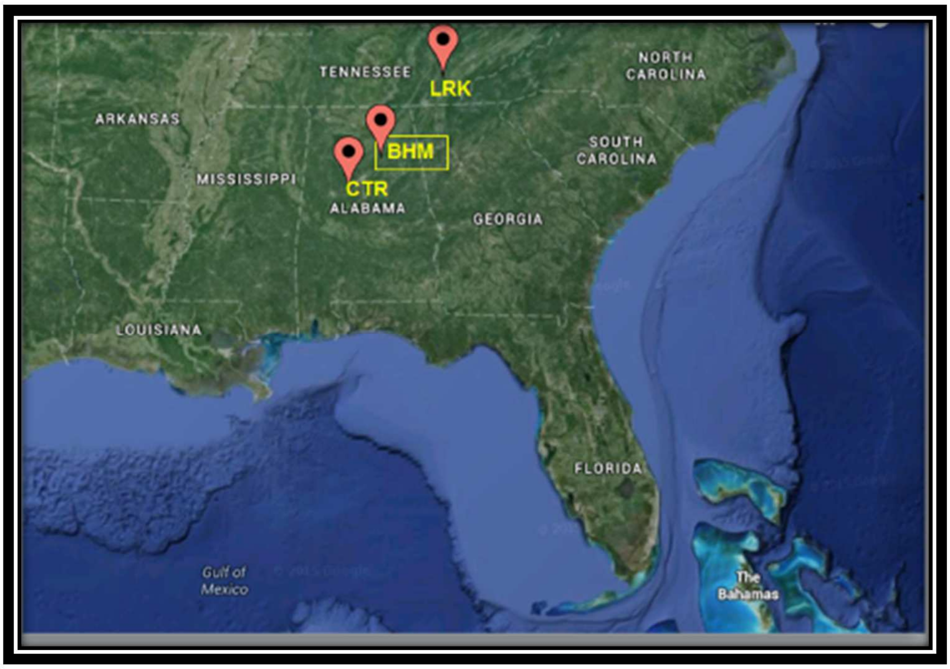

Figure S1. The locations of the three sampling sites during 2013 SOAS: BHM, CTR, and LRK. BHM was the focused site in this study.

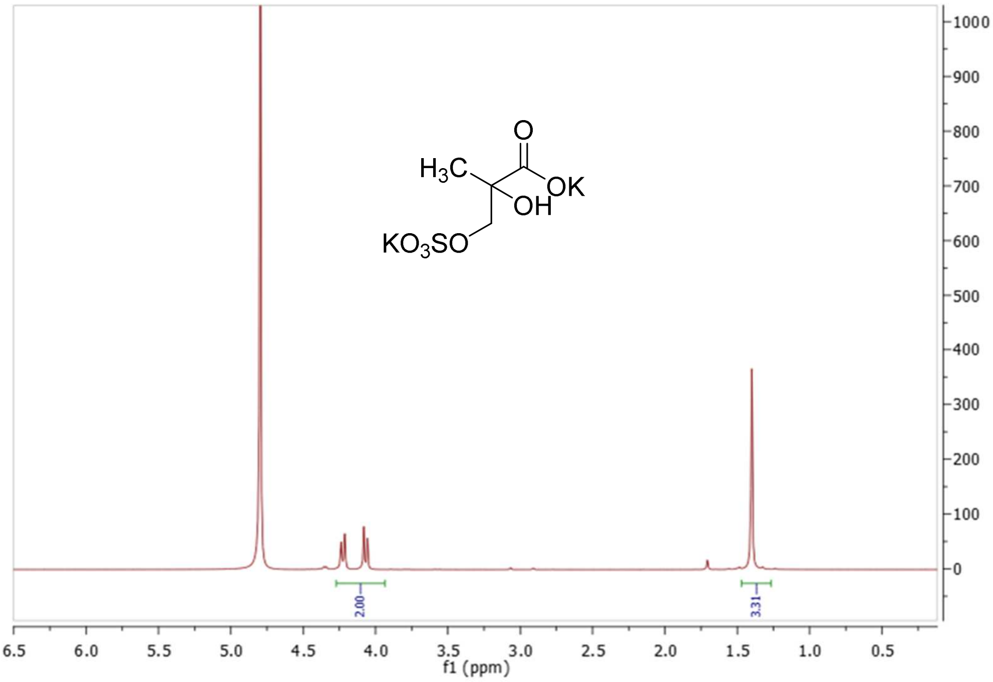

Figure S2. ^{1}H NMR (400 MHz, D_2O) of the MAE/HMML-derived OS.

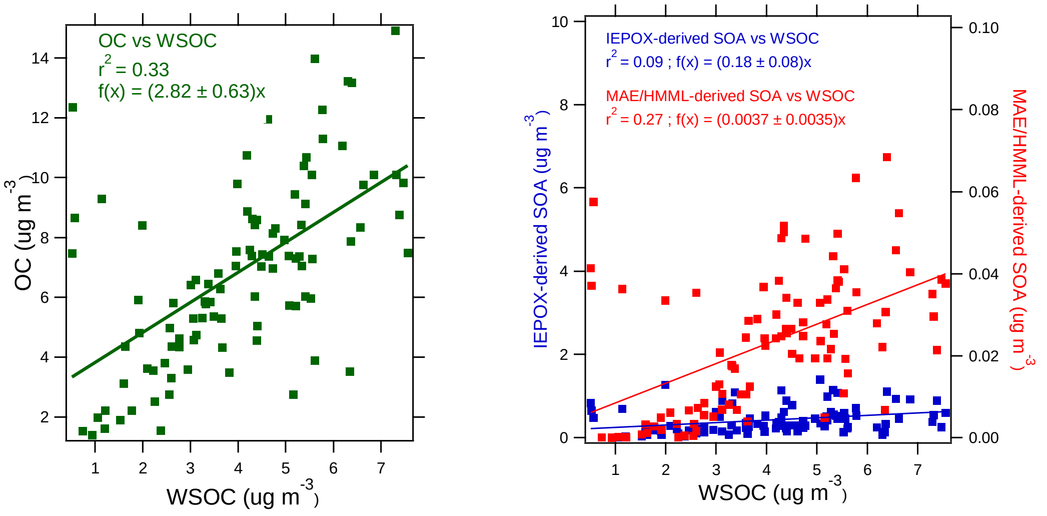

(a) (b)

Figure S3. (a) Comparison of organic carbon (OC) and water soluble organic carbon (WSOC), suggesting that 35% of OC at BHM was WSOC. (b) Comparison of IEPOX- and MAE/HMML-derived SOA tracers with WSOC, indicating that IEPOX- and MAE/HMML-derived SOA tracers explained 18 and 0.4% of the WSOC, respectively.

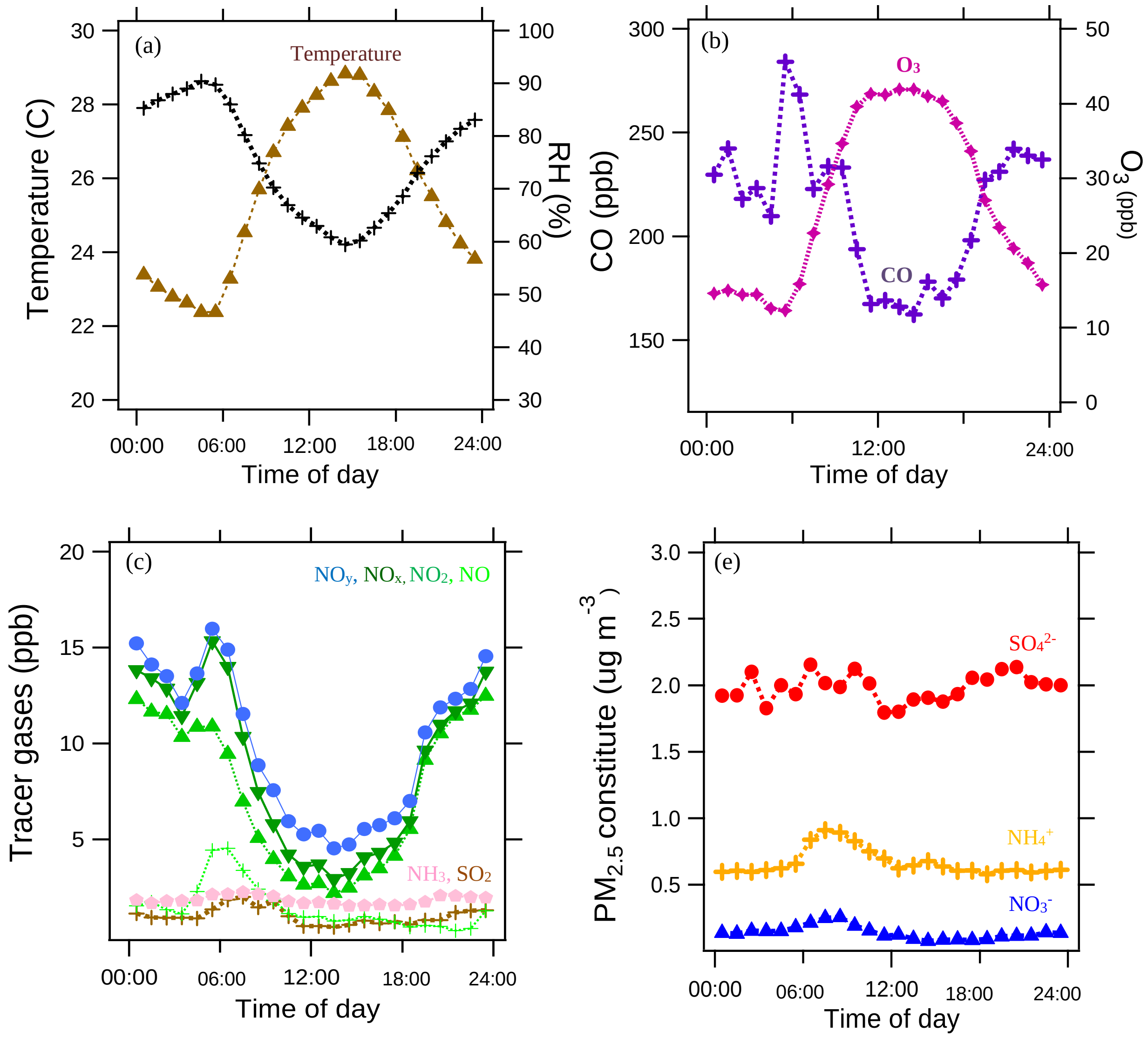

Figure S4. Diurnal variations of (a) meteorology, (b) O_3 and CO, (c) NO_y, NO, NO_2, and NO_x, and (d) $PM_{2.5}$ constituents at BHM during the 2013 SOAS campaign. High temperature and low RH were observed at 2-4 pm local time. O_3 reached its maximum, while CO dropped to its minimum in early afternoon. NO_x and NO_y were high during early morning hours and declined in the afternoon due to photochemical processes. No significant diurnal variation was observed for NH_3, SO_2, SO_4^{2-}, NH_4^+, and NO_3^-.
